# Association between modifiable lifestyle factors and telomere length: a univariable and multivariable Mendelian randomization study

**DOI:** 10.1186/s12967-024-04956-8

**Published:** 2024-02-16

**Authors:** Miao Chen, Zhen Wang, Hongfei Xu, Peng Teng, Weidong Li, Liang Ma

**Affiliations:** Department of Cardiovascular Surgery, the First Affiliated Hospital of Zhejiang University School of Medicine, Number 79 Qingchun Road, Hangzhou, China

**Keywords:** Lifestyle factors, Telomere length, Lifetime smoking, Insomnia, Sleep duration, Physical activity

## Abstract

**Background:**

Telomere length has long been recognized as a valuable biomarker of aging and is inversely correlated with chronological age. Various lifestyle factors have been implicated in telomere shortening or preservation; however, the association between lifestyle factors and telomere length remains controversial. To address this issue, we conducted a Mendelian randomization (MR) analysis to investigate the potential causal associations between multiple lifestyle factors and telomere length.

**Methods:**

Independent genetic variants strongly associated with lifestyle factors (tobacco smoking, sleep duration, insomnia, and physical activity) were selected as instrumental variables from corresponding genome-wide association studies (GWASs). Summary-level data for telomere length was obtained from a GWAS comprising 472,174 European ancestries. Univariable and multivariable MR analyses were performed to assess the relationships.

**Results:**

The genetic liability to lifetime smoking was robustly associated with shorter telomere length (odd ratio [OR]: 0.882; 95% confidence interval [CI]: 0.847–0.918). Genetically predicted insomnia was also linked to shorter telomere length (OR: 0.972; 95% CI: 0.959–0.985), while no significant association was observed between sleep duration and telomere length. Furthermore, a suggestive association was found between moderate-to-vigorous physical activity and longer telomere length (OR: 1.680; 95% CI: 1.115–2.531). In multivariable MR analyses, adjusting for potential mediators such as body mass index, type 2 diabetes, alcohol consumption, and alcohol use disorder, the associations of lifetime smoking and insomnia with telomere length remained robust.

**Conclusion:**

Our findings suggest that smoking and insomnia may contribute to telomere shortening, while physical activity may play a role in telomere length maintenance. These findings underscore the importance of managing positive risk factors and adopting a healthy lifestyle to promote telomere health.

**Supplementary Information:**

The online version contains supplementary material available at 10.1186/s12967-024-04956-8.

## Introduction

With medical and technological advances in recent decades, human life expectancy has increased worldwide and is expected to increase further in the coming years [1]. Population aging is accompanied by an increase in the incidence of age-related diseases, such as cardiovascular diseases [2], diabetes [3], and cancer [4]. For this reason, aging has become a critical public health issue in many countries. Currently, telomere length has been developed as a surrogate measure of biological age [5]. Telomeres are conserved repetitive DNA sequences, together with associated protective protein, located at the ends of cap-shaped linear chromosomes. With each cell division, telomeres naturally shorten because the DNA polymerases cannot wholly replicate the end of the lagging DNA strand. Thus, telomere shortening occurs with increasing cell age. Telomere length is partially determined by genetic factors and correlates with sex, race, and paternal age [6, 7]. It also correlates with environmental and lifestyle factors, including sleep, exercise, tobacco use and alcohol consumption [8, 9].

Lifestyle factors and telomere length have been investigated in observational studies with conflicting results. The largest cross-sectional study, conducted on 7,813 women from the Nurses’ Health Study, found that even moderate physical activity was associated with longer telomere length compared to less active individuals [10]. Similarly, a study involving 2,312 American Indian participants reported a positive association between higher levels of physical activity and longer telomeres [11]. However, a few studies with smaller sample sizes have shown no significant association between physical activity and telomere length [12–14]. The relationship between tobacco smoking and telomere length has also been investigated in observational studies with mixed findings. A dose-dependent adverse association was observed between the number of cigarettes smoked and telomere length in a study involving 8,074 participants [15]. Similarly, a longitudinal study of 5,624 participants from the Health and Retirement Study found an inverse association between the number of cigarettes smoked and telomere length, particularly in women [16]. However, a case–control study of 29 healthy smokers and 29 non-smokers reported no impact of tobacco smoking on telomere length [17]. In comparison to physical activity and smoking, few studies have specifically focused on sleep duration. Several studies have shown a significant association between shorter sleep duration and shorter telomere length [18–20]. Additionally, conflicting results have been reported regarding the effect of long sleep duration on telomere length, with some studies suggesting a U-shaped relationship or a linear effect [18, 20].

Conducting randomized controlled trials to examine the impact of lifestyle factors on telomere length is impractical due to ethical considerations. Moreover, the causal nature of the associations between the aforementioned lifestyle factors and telomere length remains uncertain in observational studies due to potential residual confounding and reverse causality issues. Consequently, alternative methods that facilitate causal inference can provide valuable insights into whether these factors represent potentially modifiable risk factors. Mendelian randomization (MR) is an analytical approach that utilizes single-nucleotide polymorphisms (SNPs) as reliable instrumental variables to assess the potential causal relationship between an exposure and an outcome. [21]. Conceptually, MR shares similarities with randomized controlled trials as genetic variants are randomly assigned during meiosis, thereby reducing concerns related to confounding and reverse causality.

In MR analyses, instrumental variables can be derived from summary statistics of extensive genome-wide association studies (GWASs) that have been conducted on lifestyle factors and telomere length. As of now, the causal relationship between the mentioned lifestyle factors and telomere length has yet to be established through MR analysis. Hence, in this study, we employed MR analysis to investigate the potential causal association between lifestyle factors (tobacco smoking, sleep duration, insomnia, and physical activity) and the telomere length.

## Method

### Study design

**Fig. 1:**
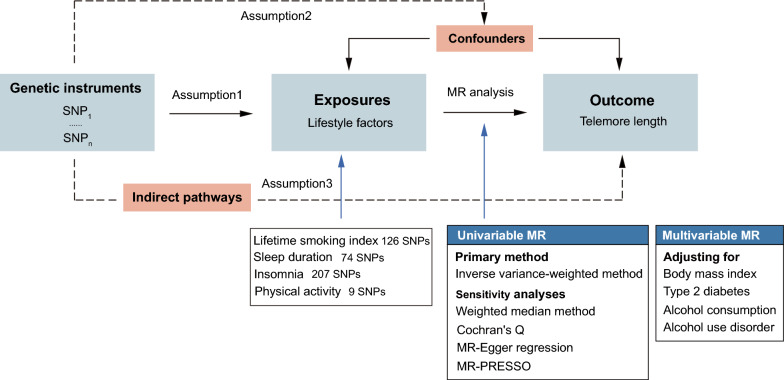
Summary of assumptions and study design in Mendelian randomization

We conducted a MR analysis utilizing SNPs as instrumental variables for the exposures under investigation. The study design is illustrated in Fig. 1. To ensure the validity of causal estimates in MR studies [21], three key assumptions need to be satisfied: First, the selected genetic variants serving as instrumental variables should be strongly associated with exposures. Second, genetic variants should not be associated with any confounders that could potentially influence the relationship between the exposures and the outcome. Third, the genetic variants should not independently affect the outcome apart from their effects on the exposures. Genetic instrument selection based on publicly available GWAS for physical activity [22], insomnia [23], sleep duration [24], and lifetime smoking [25]. Summary data for telomere length were derived from the largest-to-date GWAS [26]. Additional file 1: Table S1 provides detailed information on the data sources used in this study. The GWAS data used in this study have been made publicly available, and all ethical approval was obtained in all original studies.

### Data sources and instruments

#### Lifetime smoking

The measures of smoking used in our study were the lifetime smoking index. The SNPs associated with lifetime smoking index at the genome-wide significance level (*p*-value < 5 × 10^–8^) were extracted from a GWAS including 462,690 individuals from the UK Biobank. [25]. The lifetime smoking index aimed to capture various aspects of smoking, including smoking heaviness, duration, and initiation. It considered factors such as smoking status, age at initiation and cessation, and the number of cigarettes smoked per day. In the GWAS analysis, linear mixed models adjusted for sex and genotyping chip were conducted using BOLT-LMM. To ensure independence among the SNPs, clumping was performed with a linkage disequilibrium (LD) threshold of r^2^ = 0.01 and a distance of 10,000 kb using the TwoSampleMR package [27], utilizing the LD reference panel from the 1000 Genomes Project specifically for individuals of European descent [28]. Ultimately, 126 SNPs were identified as genetic instruments for lifetime smoking index (Additional file 1: Table S2). It is noteworthy that an increase of one standard deviation in the lifetime smoking score corresponds to a smoking history equivalent to either 20 cigarettes per day for 15 years with cessation 17 years ago or 60 cigarettes per day for 13 years with cessation 22 years ago [25].

#### Sleep duration and insomnia

The SNPs associated with sleep duration were obtained from a recent GWAS of individuals with European ancestry in the UK Biobank, which included a total of 446,118 participants [24]. Phenotypic data of sleep duration were self-reported by participants based on responses to questionnaires or interviews. Participants with sleep duration less than 3 h or more than 18 h, as well as those using sleep medication, were excluded. Sleep duration was considered both as a continuous variable and as categorical, with categories defined as short (6 h or less), normal (7 or 8 h), or long (9 h or more) sleep duration [24]. Genetic association analysis was performed using BOLT-LMM linear mixed models and an additive genetic model adjusted for age, sex, ten principal components of ancestry, and genetic correlation matrix. The GWAS identified 78 SNPs that reached genome-wide significance (*p*-value < 5 × 10^–8^), collectively accounting for 0.69% of the variance in sleep duration. Following clumping based on a LD threshold of r^2^ = 0.01 and a distance of 10 000 kb, a total of 74 independent SNPs were identified as instrumental variables for sleep duration (Additional file 1: Table S3). Additionally, separate GWAS for short (< 7 h) and long sleep duration (≥ 9 h) revealed 26 and 7 significant SNPs, respectively (Additional file 1: Tables S4 and S5).

We utilized SNPs associated with insomnia from a GWAS that involved 1,331,010 individuals of European ancestry (397,959 cases and 933,057 controls) [23]. Insomnia was defined by self-reported insomnia symptoms based on responses to questionnaires in a touchscreen device or an online survey. Genetic association analysis was conducted using PLINK logistic regression and an additive genetic model adjusted for age, sex, ten principal components, and genotyping array. The GWAS identified 248 significant SNPs, which collectively accounted for approximately 2.6% of the variance in insomnia. Among these, 207 independent SNPs were selected as instrumental variables following clumping based on the aforementioned strategy (Additional file 1: Tables S6). Sensitivity analysis excluding shift workers or individuals who reported using psychiatric medications or have psychiatric illnesses revealed a strong association with the primary GWAS [23].

#### Physical activity

We obtained SNPs associated with physical activity from a recent GWAS [22]. Moderate physical activity (MPA) and vigorous physical activity (VPA) were defined based on self-reported information collected through a touchscreen questionnaire. Moderate-to-vigorous physical activity (MVPA) was calculated as the combination of MPA and VPA, following the previously described methodology [29]. Participants with missing physical activity data or reporting more than 16 h of physical activity were excluded from the analysis. To adjust the phenotype, the GWAS employed regression analysis, where the dependent physical activity variables were regressed on several independent variables, including age, sex, genotyping chip, the first ten genomic principal components, center, and season (month) at the center visit or during accelerometer wear. Within the MVPA category, the GWAS identified nine significantly associated SNPs using data from 377,234 individuals (Additional file 1: Table S7), explaining approximately 0.09% of the variance in exposure. Moreover, five loci showed significant associations with vigorous physical activity (VPA), and six loci were significantly associated with strenuous sports or other exercises (SSOE) (Additional file 1: Tables S8 and S9).

#### Telomere length

Summary-level data for the exposure-associated SNPs with telomere length came from the largest GWAS in 472,174 UK Biobank participants [26]. Participants between the ages of 45 and 69 were recruited and extracted DNA from peripheral blood leukocytes using an automated process or manual method [30]. Leukocyte telomere length is measured as the ratio of telomere repeats copy number relative to that of a single copy gene using the qPCR method. Samples were re-run later to assess the measurements’ stability and repeatability. GWAS was performed using BOTL-LMM adjusting for age, sex, genotype array, and the first ten principal components.

#### Potential confounders

The effects of genetic variants on body mass index were obtained from publicly available summary statistics [31]. The GWAS meta-analysis included 339,224 individuals from 125 studies. Body mass index was measured or self-reported weight in kg per height in meters squared. The summary statistics of alcohol consumption were derived from the largest GWAS, including 1.2 million individuals of European ancestry [32]. Alcohol consumption was measured as weekly drinks (n = 941,280 individuals). We obtained the genetic variants for alcohol use disorder from the IEU Open GWAS Project (https://gwas.mrcieu.ac.uk), which included 12,204 cases and 206,588 controls.

#### Testing instrument strength and statistical power

F-statistics were used to evaluate the instrument strength by using the formula: F = R^2^ × (N − 1)/(1 − R^2^) [33], where N indicates the GWAS sample size. R^2^ is calculated using the formula: R^2^ = 2 × EAF × (1 − EAF) × beta^2^, where EAF is the effect allele frequency, and beta is the estimated effect on exposure [34]. The risk of weak instrument bias in MR analysis is relatively low when the F-statistics ≥ 10 [35]. We used an online tool (https://shiny.cnsgenomics.com/mRnd/) to estimate the power of our study based on the GWAS sample size and the variance explained by genetic instruments for the exposure [36]. The post hoc power calculations for our main IVW analyses were presented in Additional file 1: Table S10.

### Statistics analyses

For primary MR analysis, we used inverse variance-weighted (IVW) based on a multiplicative random-effects model as the primary method. The Wald ratio estimate was calculated by dividing the effect size (β per additional risk allele) for the SNP-telomere length association by the corresponding coefficient for the SNP-exposure association [37]. Subsequently, we combined the estimates for each SNP to derive a single causal estimate for each exposure.

To assess the robustness of our findings, we conducted various sensitivity analyses to evaluate the impact of potential pleiotropic effects on our causal estimates. In addition to the IVW method, we employed the weighted median method. The weighted median estimate of a causal effect remains consistent as long as at least 50% of the genetic variants are valid instrumental variables [38]. Cochran’s Q and I^2^ statistics were used to evaluate the heterogeneity produced by different instrumental variables in the analysis [39]. A *p*-value of Cochran’s Q < 0.05 indicated the presence of heterogeneity, and the I^2^ statistic reflected the level of heterogeneity (I^2^ < 25%: small heterogeneity; I^2^ = 25–75%: moderate heterogeneity; I^2^ > 75%: large heterogeneity). When heterogeneity was detected, a random-effects IVW analysis was more appropriate. Additionally, we performed the MR-Egger intercept test to detect the potential presence of horizontal pleiotropy, with a deviation from zero (*p*-value < 0.05) indicating evidence of directional pleiotropic bias [40]. In the presence of horizontal pleiotropy, the slope coefficient from the MR-Egger regression provided a reliable estimate of the causal effect. MR pleiotropy residual sum and outlier (MR-PRESSO) was used to identify and correct horizontal pleiotropic outliers [41]. If any outlying SNPs were identified, we reassessed the effect estimates by excluding them from the exposure instruments.

Given the possibility of confounding factors influencing the relationship between these exposures and telomere length, we performed multivariable MR analyses to assess the direct effect of exposures on telomere length whilst accounting for potential confounding effects of body mass index, type 2 diabetes, alcohol consumption, and alcohol use disorder. Additionally, considering the positive association between tobacco smoking and insomnia [23], we also conducted multivariable MR analyses to adjust for the lifetime smoking index and insomnia in relation to each other.

In our complementary analysis, we employed statistical methods similar to those described above to investigate the causal relationships between short sleep duration, long sleep duration, VPA, and SSOE with telomere length.

Statistical significance was defined as a *p*-value < 0.05. To account for multiple exposures, we adjusted the significance threshold using Bonferroni correction, resulting in a *p*-value of < 0.0125 (= 0.05/4 exposures) considered statistically significant. All analyses were conducted using R version 3.6.1 with the TwoSampleMR (version 0.5.5), MendelianRandomization (version 0.4.3), and MRPRESSO (version 1.0) packages.

## Results

### Lifetime smoking index

**Fig. 2:**
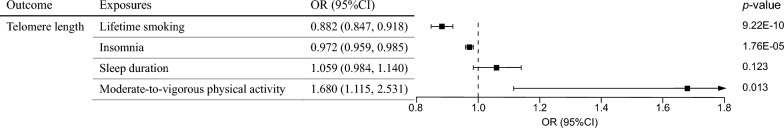
Mendelian randomization association of genetic liability to lifestyle factors with telomere length. OR, odds ratio; CI, confidence interval. Estimates are from the random-effects inverse variance weighted method. Significant at the Bonferroni-corrected threshold of *p*-value < 0.0125

**Fig. 3:**
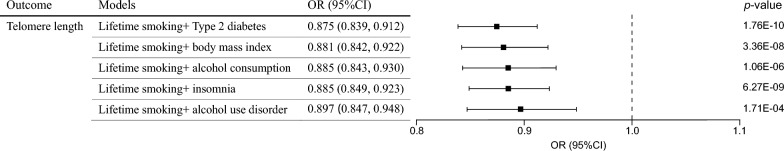
Multivariable mendelian randomization was performed to estimate the direct effect of the association of lifetime smoking with telomere length after accounting for other confounders

The genetic liability to lifetime smoking was strongly associated with shorter telomere length (OR: 0.882; 95% CI: 0.847–0.918; *p* = 9.22 × 10^–10^) (Fig. 2). Consistent findings were obtained from the weight median method, as shown in Additional file 1: Table S11. Evaluation of heterogeneity using Cochran’s Q and I^2^ statistics revealed moderate variability across SNPs (Additional file 1: Table S12). Furthermore, the intercept of MR-Egger intercept indicated no evidence of directional pleiotropy (0.001; *p* = 0.532) (Additional file 1: Table S12). Outlier detection using MR-PRESSO identified three SNPs, but upon removal of these variants, the causal estimate remained unchanged (Additional file 1: Table S13). In the multivariable analysis adjusting for body mass index, type 2 diabetes, alcohol consumption, alcohol use disorder, the associations between genetic susceptibility to lifetime smoking and telomere length did not diminish and remained statistically significant (Fig. 3). Considering the observed genetic association between smoking and insomnia, we additionally accounted for insomnia in the multivariable MR analysis. Notably, the inclusion of insomnia as a covariate did not alter the association between lifetime smoking and telomere length (Fig. 3).

### Insomnia

**Fig. 4:**
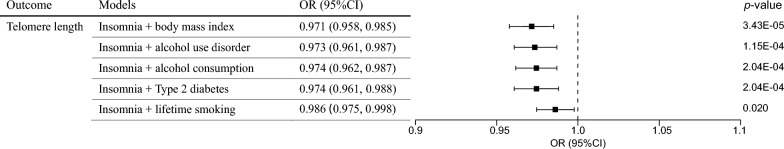
Multivariable mendelian randomization was performed to estimate the direct effect of the association of insomnia with telomere length after accounting for other confounders

Genetic predisposition to insomnia was significantly associated with shorter telomere length, as evident in the random-effects IVW analysis (OR: 0.972; 95% CI: 0.959–0.985; *p* = 1.76 × 10^–5^) and weight median analysis (OR: 0.980; 95% CI: 0.968–0.991; *p* = 5.34 × 10^–4^) (Fig. 2 and Additional file 1: Table S11). Cochran’s Q and I^2^ statistics revealed moderate heterogeneity across SNPs (Additional file 1: Table S12). The intercept of the MR-Egger was close to zero (-0.001; *p* = 0.626), suggesting the absence of directional pleiotropy (Additional file 1: Table S12). Through MR-PRESSO, nine SNPs were identified as potential outliers, yet exclusion of these variants did not alter the results (Additional file 1: Table S13). Moreover, even after adjusting for potential confounders such as body mass index, type 2 diabetes, alcohol consumption, and alcohol use disorder, the association between genetic liability to insomnia and telomere length remained statistically significant (Fig. 4). However, upon further adjustment for lifetime smoking, the effect sizes of the association slightly attenuated and did not reach statistical significance after Bonferroni correction (OR: 0.986; 95% CI: 0.975–0.998; *p* = 0.020) (Fig. 4).

### Sleep duration

There was no significant association found between genetically predicted sleep duration and telomere length (OR: 1.059; 95% CI: 0.984–1.140; *p* = 0.123) (Fig. 2). The weighted median method yielded consistent estimates with the primary analysis (Additional file 1: Table S11). Notably, there was substantial heterogeneity across SNPs for sleep duration, as indicated by significant Cochran’s Q and I^2^ statistics (Additional file 1: Table S12). Moreover, no evidence of directional pleiotropy was observed (MR-Egger intercept: 0.001; *p* = 0.605). The estimate remained unchanged even after excluding five outlier SNPs identified by the MR-PRESSO method (Additional file 1: Table S13).

In the complementary analyses, we employed similar statistical methods to investigate the causal effects of short and long sleep duration on telomere length. The findings suggested a potential association between short sleep duration and telomere length (OR: 0.959; 95% CI: 0.923–0.996; *p* = 0.032). However, genetically predicted long sleep duration showed no association with telomere length (OR: 1.004; 95% CI: 0.959–1.051; *p* = 0.865).

### Physical activity

The results revealed a suggested association between genetically predicted MVPA and longer telomere length (OR: 1.680; 95% CI: 1.115–2.531; *p* = 0.013) (Fig. 2). However, the estimate obtained from the weighted median method showed inconsistency with the primary analysis (Additional file 1: Table S11). The presence of high heterogeneity among these SNPs was assessed using Cochran’s Q and overall I^2^ statistics (Additional file 1: Table S12). The MR-Egger intercept test detected a possible directional pleiotropy (-0.033; *p* = 0.007). After accounting for pleiotropy in the MR-Egger analysis, the genetic liability to MVPA exhibited a positive association with telomere length (OR 11.934; 95% CI, 4.167- 34.180; *p* = 0.002) (Additional file 1: Table S11). By applying the MR-PRESSO method, six SNPs were identified as outliers, and even after excluding these SNPs, a suggested association between genetically predicted MVPA and longer telomere length persisted (OR: 2.482; 95% CI: 1.171–5.263; *p* = 0.018) (Additional file 1: Table S13).

Complementary analyses were conducted to investigate the association between VPA and SSOE with telomere length. In contrast to MVPA, no causal associations were observed between genetically predicted VPA (OR: 0.988; 95% CI: 0.945–1.034; *p* = 0.606), SSOE (OR: 1.019; 95% CI: 0.963–1.080; *p* = 0.511), and telomere length.

## Discussion

### Principal findings

In this MR study, we utilized large-scale human genetics data to investigate the potential causal relationships between physical activity, lifetime smoking, sleep duration, insomnia, and telomere length. By leveraging data from multiple large-scale GWASs, we were able to incorporate a larger number of cases involving lifestyle factors and telomere length compared to previous observational studies. Notably, we observed robust associations between lifetime smoking, insomnia, and shorter telomere length. While suggestive associations were found between MVPA and longer telomere length, no significant association was observed between sleep duration and telomere length. Importantly, the associations of lifetime smoking and insomnia with telomere length remained significant even after adjusting for confounding factors such as alcohol consumption, alcohol use disorder, body mass index, and type 2 diabetes.

### Previous researches

The association between lifetime smoking and shorter telomere length was strong and likely to be causal, as indicated by consistent estimates across the employed MR methods. Furthermore, even after adjusting for potential confounding factors, the association between smoking and telomere length remained significant, providing further support for the plausibility of the hypothesis. Notably, findings from prospective observational studies yielded similar results. For instance, a comprehensive longitudinal study involving 5,624 participants over a 16-year period revealed a consistent link between smoking and reduced telomere length, albeit with variations by gender [16]. Another study [42] reported a negative association between smoking and telomere length in older adults, indicating a potential difference of 73 base pairs between smoking and non-smoking individuals. Additionally, a large longitudinal study encompassing 1,356 individuals aged 30–70 suggested that smoking status was associated with both telomere length attrition and baseline telomere length, further supporting the adverse impact of smoking on telomeres [43].

Deriving a causal relationship between sleep duration and telomere length is not so straightforward. In this MR study, no compelling evidence was found for a direct causal effect of continuous sleep duration on telomere length. However, when utilizing SNPs associated with short and long sleep durations to estimate causal effects, the results suggested a link between genetically predicted short sleep duration (< 7 h) and shorter telomere length, while no significant association was observed for long sleep duration (≥ 9 h). Observational studies investigating the association between sleep duration and telomere length have yielded conflicting findings. Zhao et al. [44] conducted a study that revealed a positive correlation between telomere length and daily sleeping time. In contrast, another study found an association between long sleep duration and shorter telomere length, even after adjusting for factors such as sex, age, and body mass index. However, two cross-sectional studies indicated that individuals with shorter sleep duration exhibited decreased telomere length [45, 46]. Insomnia, primarily a mental disorder characterized by poor sleep quality and shortened sleep duration, was also explored in our MR analyses. The results further supported a connection between genetically predicted insomnia and shorter telomere length. In line with our findings, an observational study involving 925 individuals reported a relationship between insomnia disorder (OR = 2.654, 95% CI = 1.025–6.873) and shortened telomere length [20].

Drawing definitive conclusions about the causal role of MVPA in telomere length is currently not possible. However, our MR analyses revealed a suggestive positive association between genetic predisposition to MVPA and telomere length. The relatively wide 95% confidence interval for the estimate may be attributed to the small variance explained (0.09%) by MVPA. Furthermore, our additional analyses did not indicate any associations between VPA, SSOE, and telomere length. Several large cross-sectional studies have reported a positive association between physical activity and telomere length [47–49]. Additionally, two longitudinal studies in elderly subjects demonstrated a positive relationship between physical activity and telomere length [50, 51]. However, the largest longitudinal study, involving 4,576 participants from the Copenhagen City Heart Study, found no association between changes in physical activity and telomere length over a ten-year follow-up period [52]. Results from intervention studies have also been mixed. Most trials with small sample sizes reported that participants in active physical activity interventions showed increased telomere length compared to control groups [53–55]. In contrast, a trial involving 439 women comparing telomere length after a 12-month physical activity intervention did not find any significant differences [56]. The optimal type and amount of physical activity required to maintain telomere length remain a topic of debate, indicating the need for further research.

### Potential mechanisms

The precise pathophysiological mechanisms underlying the association between lifestyle factors and telomere length remain incompletely understood. However, oxidative stress and inflammation have been identified as potential explanations for this relationship [57, 58]. Both tobacco smoking and insomnia have been shown to increase the levels of inflammatory cytokines and pro-oxidants, thereby disrupting the balance between inflammatory and oxidative stress pathways [59, 60]. Conversely, physical activity has been found to reduce inflammation and oxidative stress by decreasing the production of reactive oxygen species, tumor necrosis factor α, C-reactive protein, and interleukin-6 [61, 62]. Moreover, exercise has been associated with increased plasma levels of irisin and insulin-like growth factor 1, which are directly linked to telomere length [63–65].

### Strengths and limitations

This study possessed several strengths. The primary strength lies in the MR design, which mitigates confounding biases often present in observational studies. By utilizing genetic associations obtained from available GWAS as instrumental variables for different lifestyle factors, we were able to address potential horizontal pleiotropy. Additionally, we conducted numerous sensitivity analyses, including multivariate MR, to assess the robustness of our findings. Another strength is the inclusion of participants of European ancestry exclusively, thereby minimizing the influence of population stratification bias.

Nevertheless, several limitations were observed in this study. Firstly, we cannot entirely dismiss the possibility that the genetic variants associated with lifestyle factors also influence telomere length through alternative causal pathways. However, our robust associations persisted even after adjusting for major genetically related risk factors such as alcohol use and body mass index in multivariate MR analyses. Another limitation is our inability to explore potential nonlinear associations between physical activity and telomere length. Despite the large sample size for physical activity, the genetic instruments explaining the variation in exposure were limited, leading to wide 95% confidence intervals. Thirdly, the genetic variants for both exposures and outcomes were measured in the same individuals within the UK Biobank, which is known as one-sample MR. Although this ensures identical demographic characteristics (e.g., age, sex, socioeconomic background), it can inflate false positive rates (type 1 error). Fourthly, it is important to note that while MR studies provide valuable insights into the causal effects of lifetime exposures [66], they are limited in capturing variations attributed to non-genetic exposures that occur throughout an individual’s lifetime. Lastly, the generalizability of our findings may be limited to populations of European ancestry, and caution should be exercised when extrapolating the results to other racial groups. For instance, tobacco smoking is likely to be a potent risk factor across all racial groups.

## Conclusion

The current MR study provided evidence supporting the association between lifetime smoking and insomnia with shorter telomere length, while no causal relationship was found between sleep duration and telomere length. However, further investigation is required to explore the potential causal role of physical activity in promoting longer telomere length, particularly through larger-scale MR analyses in the future. Consequently, adopting a healthy and active lifestyle may have potential health benefits.

### Supplementary Information

**Additional file 1. Table S1.** Descriptive information of the datasets included in the analyses. **Table S2.** Associations of single nucleotide polymorphisms for lifetime smoking index. **Table S3.** Associations of single nucleotide polymorphisms for continuant sleep duration. **Table S4.** Associations of single nucleotide polymorphisms for short sleep duration. **Table S5.** Associations of single nucleotide polymorphisms for long sleep duration.**Table S6.** Associations of single nucleotide polymorphisms for insomnia. **Table S7.** Associations of single nucleotide polymorphisms for moderate-to-vigorous physical activity. **Table S8.** Associations of single nucleotide polymorphisms for vigorous physical activity. **Table S9.** Associations of single nucleotide polymorphisms for strenuous sports or other exercises. **Table S10.** Post-hoc power calculations for our main IVW analyses on lifestyle factors and telomere length. **Table S11.** Estimates for the association between lifestyle factors and telomere length. **Table S12.** Heterogeneity and MR-Egger test for Horizontal pleiotropy. **Table S13.** Associations between genetic liability to lifestyle factors and telomere length following exclusion of outlier SNPs identified by MR-PRESSO.

## Data Availability

Our study used publicly available summary-level data of GWAS. The summary statistics for lifetime smoking is available at the website https://doi.org/10.5523/bris.10i96zb8gm0j81yz0q6ztei23d. The summary statistics for sleep duration is available at the website https://sleep.hugeamp.org/downloads.html. The summary statistics for insomnia is available at the website https://ctg.cncr.nl/software/summary_statistics. The summary statistics for telomere length is available at the website https://figshare.com/s/caa99dc0f76d62990195 (Exclude 23and me). The summary statistics for alcohol use is available at the website https://genome.psych.umn.edu/index.php/GSCAN. The summary statistics of GWAS for type 2 diabetes, body mass index, and alcohol use disorder can be accessed at IEU OpenGWAS project (https://gwas.mrcieu.ac.uk/); Each IDs were presented in Additional file 1: Table S1. The code used in the current study can be obtained from the additional information.
