# Supplementary material for: Association between modifiable lifestyle factors and telomere length: a univariable and multivariable Mendelian randomization study
Source: J Transl Med. 2024 Feb 16;22:160. doi: 10.1186/s12967-024-04956-8 (PMC10870665; doi:10.1186/s12967-024-04956-8)
Supplement: Supplementary file 1 — Additional file 1. Table S1. Descriptive information of the datasets included in the analyses. Table S2. Associations of single nucleotide polymorphisms for lifetime smoking index. Table S3. Associations of single nucleotide polymorphisms for continuant sleep duration. Table S4. Associations of single nucleotide polymorphisms for short sleep duration. Table S5. Associations of single nucleotide polymorphisms for long sleep duration.Table S6. Associations of single nucleotide polymorphisms for insomnia. Table S7. Associations of single nucleotide polymorphisms for moderate-to-vigorous physical activity. Table S8. Associations of single nucleotide polymorphisms for vigorous physical activity. Table S9. Associations of single nucleotide polymorphisms for strenuous sports or other exercises. Table S10. Post-hoc power calculations for our main IVW analyses on lifestyle factors and telomere length. Table S11. Estimates for the association between lifestyle factors and telomere length. Table S12. Heterogeneity and MR-Egger test for Horizontal pleiotropy. Table S13. Associations between genetic liability to lifestyle factors and telomere length following exclusion of outlier SNPs identified by MR-PRESSO. [file 12967_2024_4956_MOESM1_ESM.pdf]

Association between modifiable lifestyle factors and telomere length: A Mendelian randomization study

Supplementary Material

Table 1. Descriptive information of the datasets included in the analyses.

GWAS	Phenotype	Participants	Ancestry	Used in MR	ID in IGD*	Web link for data source
Wootton, R. E. 2021	Lifetime smoking	462,690 individuals	European	Exposure	NA.	https://doi.org/10.5523/bris.10i96zb8gm0j81yz0q6ztei23d
Jansen, P. R. 2019	Insomnia	1,331,010 individuals	European	Exposure	NA.	https://ctg.cncr.nl/software/summary_statistics
Dashti, H. S. 2019	Sleep duration	446,118 individuals	European	Exposure	NA.	https://sleep.hugeamp.org/downloads.html
Dashti, H. S. 2019	Short Sleep duration	106,192 cases<br>305,742 controls	European	Exposure	NA.	https://sleep.hugeamp.org/downloads.html
Dashti, H. S. 2019	Long Sleep duration	34,184 cases<br>305,742 controls	European	Exposure	NA.	https://sleep.hugeamp.org/downloads.html
Klimentidis, Y. C. 2018	Moderate-to-vigorous physical activity	377,234 individuals	European	Exposure	NA.	NA.
Klimentidis, Y. C. 2018	Vigorous physical activity	98,060 cases;<br>162,995 controls	European	Exposure	NA.	NA.
Klimentidis, Y. C. 2018	Strenuous sports or other exercises	124,842 cases<br>225,650 controls	European	Exposure	NA.	NA.
Codd, V. 2021	Telomere length (Exclude 23and me)	472,174 individuals	European	Outcome	NA.	https://figshare.com/s/caa99dc0f76d62990195
DIAGRAM 2014	Type 2 diabetes	26,488 cases<br>83,964 controls	Multi-ancestry	Confounder	ieu-a-23	https://gwas.mrcieu.ac.uk/
Locke AE 2015	Body mass index	339,224 individuals	Multi-ancestry	Confounder	ieu-a-2	https://gwas.mrcieu.ac.uk/
GSCAN 2019	Alcohol use	335,394 individuals	European	Confounder	ieu-b-73	https://genome.psych.umn.edu/index.php/GSCAN
AUD_SWEDISH 2021	Alcohol use disorder	12,204 cases<br>206,588 controls	European	Confounder	finn-b-AUD_SWEDISH	https://gwas.mrcieu.ac.uk/

*IGD: The IEU GWAS database (https://gwas.mrcieu.ac.uk/)

Table 2. Associations of single nucleotide polymorphisms for lifetime smoking index.

SNP	Chr	Position	EA	NEA	EAF	Beta	SE	*p*-value	N*	F-statistic	Outcome	
											Beta	SE
rs11210229	1	73860028	A	G	0.384	0.017	0.002	2.00E-16	462,690	63.3	-0.0059	0.0020
rs549845	1	44076469	G	A	0.301	0.016	0.002	8.30E-14	462,690	49.8	0.0072	0.0021
rs10922907	1	91193049	A	T	0.451	0.015	0.002	3.00E-13	462,690	51.6	-0.0008	0.0021
rs7553348	1	75005067	G	A	0.438	0.014	0.002	5.20E-12	462,690	44.7	-0.0019	0.0021
rs7528604	1	66407352	G	A	0.566	0.014	0.002	5.70E-12	462,690	44.6	-0.0016	0.0021
rs1933270	1	49977965	T	G	0.364	0.013	0.002	1.50E-10	462,690	36.2	0.0014	0.0021
rs7519626	1	99514554	C	T	0.324	0.012	0.002	1.20E-08	462,690	29.2	0.0001	0.0021
rs9435340	1	107593201	T	A	0.344	0.012	0.002	1.20E-08	462,690	30.1	-0.0049	0.0020
rs4949465	1	32178489	T	C	0.87	-0.017	0.003	1.70E-08	462,690	30.2	-0.0018	0.0021
rs10918701	1	162090536	G	A	0.372	0.012	0.002	2.10E-08	462,690	31.1	0.0076	0.0051
rs1193237	1	7526486	G	C	0.439	-0.011	0.002	2.80E-08	462,690	27.6	0.0035	0.0023
rs1931263	1	96175101	G	T	0.51	-0.011	0.002	4.00E-08	462,690	28.0	0.0036	0.0033
rs2890772	2	146175106	G	T	0.413	-0.02	0.002	2.10E-22	462,690	89.8	-0.0016	0.0021
rs62155874	2	162090536	A	G	0.873	-0.024	0.003	5.20E-16	462,690	59.1	-0.0006	0.0028
rs2867112	2	651349	T	G	0.835	0.021	0.003	4.80E-15	462,690	56.2	-0.0013	0.0024
rs7569203	2	45154418	A	C	0.689	-0.016	0.002	7.40E-13	462,690	50.8	-0.0016	0.0020
rs12623702	2	202885506	A	G	0.613	-0.014	0.002	7.70E-12	462,690	43.0	0.0010	0.0024
rs4671357	2	60136176	T	C	0.519	-0.014	0.002	1.10E-11	462,690	45.3	0.0068	0.0020
rs4473348	2	182073742	A	T	0.25	-0.015	0.002	6.40E-11	462,690	39.0	-0.0012	0.0020
rs2678670	2	104469564	A	T	0.486	0.013	0.002	3.10E-10	462,690	39.1	-0.0032	0.0021
rs62135536	2	44326028	C	T	0.968	0.035	0.006	8.00E-10	462,690	35.1	0.0035	0.0022
rs3811038	2	113240183	T	C	0.724	-0.014	0.002	8.90E-10	462,690	36.2	0.0026	0.0022
rs359243	2	60475509	T	C	0.393	-0.013	0.002	9.50E-10	462,690	37.3	0.0034	0.0021
rs13016665	2	57995348	C	A	0.577	-0.012	0.002	1.80E-09	462,690	32.5	0.0077	0.0022
rs3769949	2	166199284	T	A	0.528	-0.012	0.002	2.50E-09	462,690	33.2	0.0085	0.0024
rs13009008	2	174043233	A	G	0.328	0.012	0.002	4.60E-09	462,690	29.4	0.0006	0.0021
rs6741228	2	22548774	T	C	0.433	0.011	0.002	1.60E-08	462,690	27.5	-0.0030	0.0021
rs62175972	2	161362830	T	C	0.966	0.031	0.006	1.70E-08	462,690	29.2	0.0051	0.0020
rs6778080	3	49317338	T	C	0.267	0.016	0.002	1.30E-12	462,690	46.4	0.0086	0.0032
rs326341	3	107811142	G	A	0.525	0.014	0.002	1.20E-11	462,690	45.2	0.0037	0.0021
rs421983	3	84892866	T	C	0.519	0.013	0.002	3.30E-10	462,690	39.0	0.0027	0.0026
rs6779302	3	16859710	G	T	0.633	-0.013	0.002	1.20E-09	462,690	36.3	0.0023	0.0028
rs9842947	3	157412246	C	T	0.326	-0.013	0.002	3.10E-09	462,690	34.4	0.0060	0.0023
rs775758	3	77582005	A	T	0.433	0.012	0.002	1.10E-08	462,690	32.7	-0.0027	0.0032
rs73220544	3	131074511	A	C	0.842	-0.016	0.003	1.50E-08	462,690	31.5	-0.0018	0.0022
rs17576594	4	147952241	G	A	0.724	0.016	0.002	1.70E-12	462,690	47.3	-0.0004	0.0021

rs72678864	4	112422145	G	A	0.829	0.018	0.003	1.60E-11	462,690	42.5	0.0018	0.0020
rs317021	4	35418368	T	A	0.814	-0.017	0.003	1.10E-10	462,690	40.5	-0.0032	0.0021
rs624833	4	2881256	T	G	0.695	0.013	0.002	6.60E-10	462,690	33.2	0.0016	0.0025
rs61796681	4	23678196	A	T	0.912	-0.019	0.004	4.20E-08	462,690	26.8	0.0006	0.0021
rs986391	5	166993972	G	A	0.367	0.016	0.002	9.40E-15	462,690	55.0	-0.0023	0.0023
rs329120	5	133861756	C	T	0.581	0.014	0.002	6.30E-12	462,690	44.2	0.0004	0.0024
rs13153393	5	167604213	A	G	0.884	-0.02	0.003	2.50E-10	462,690	38.0	-0.0031	0.0020
rs11948770	5	13246336	T	C	0.768	-0.015	0.002	4.90E-10	462,690	37.1	-0.0049	0.0023
rs71627581	5	43161351	G	A	0.889	0.019	0.003	1.60E-09	462,690	33.0	0.0016	0.0020
rs10052591	5	50812738	T	C	0.573	0.012	0.002	2.10E-09	462,690	32.6	0.0030	0.0020
rs4957528	5	106420589	A	C	0.208	-0.015	0.002	4.20E-09	462,690	34.3	0.0040	0.0022
rs245774	5	170530930	A	G	0.272	-0.013	0.002	7.40E-09	462,690	31.0	0.0020	0.0021
rs4571506	5	87756918	C	T	0.54	0.011	0.002	1.50E-08	462,690	27.8	-0.0026	0.0027
rs2080870	5	60388313	A	T	0.258	0.012	0.002	4.90E-08	462,690	25.5	0.0025	0.0020
rs7766610	6	111707821	C	A	0.183	0.018	0.003	2.20E-12	462,690	44.8	-0.0010	0.0037
rs6935954	6	26255451	A	G	0.421	0.014	0.002	8.20E-12	462,690	44.2	0.0016	0.0026
rs12202536	6	67475273	A	G	0.513	-0.012	0.002	2.80E-09	462,690	33.3	-0.0017	0.0020
rs2894808	6	52861990	T	A	0.922	-0.022	0.004	3.50E-09	462,690	32.2	-0.0093	0.0020
rs2254710	6	37477000	C	A	0.236	0.013	0.002	3.50E-08	462,690	28.2	0.0029	0.0022
rs10226228	7	32315613	A	G	0.63	-0.016	0.002	2.00E-15	462,690	55.2	0.0027	0.0021
rs2401924	7	115057862	G	C	0.502	0.015	0.002	2.70E-14	462,690	52.1	0.0001	0.0021
rs7807019	7	117543063	A	G	0.54	-0.015	0.002	6.70E-14	462,690	51.7	0.0079	0.0025
rs1922018	7	3560401	C	T	0.364	0.014	0.002	3.00E-12	462,690	42.0	0.0000	0.0023
rs10282292	7	111092478	C	T	0.362	0.013	0.002	5.90E-10	462,690	36.1	0.0005	0.0021
rs11768481	7	96629103	C	A	0.666	0.013	0.002	9.90E-10	462,690	34.8	0.0009	0.0022
rs6962772	7	99081730	A	G	0.846	0.016	0.003	7.80E-09	462,690	30.9	0.0022	0.0020
rs4731925	7	132664757	C	T	0.316	-0.012	0.002	2.60E-08	462,690	28.8	0.0032	0.0020
rs6957896	7	132309592	C	T	0.503	-0.011	0.002	4.50E-08	462,690	28.0	0.0053	0.0023
rs11783093	8	27425349	C	T	0.839	0.023	0.003	1.20E-16	462,690	66.1	-0.0049	0.0021
rs35169606	8	9604066	T	G	0.612	0.013	0.002	1.20E-09	462,690	37.1	0.0006	0.0020
rs2062882	8	91839576	G	A	0.587	-0.012	0.002	1.10E-08	462,690	32.3	0.0003	0.0022
rs72674867	8	95578201	A	T	0.765	0.013	0.002	3.80E-08	462,690	28.1	0.0042	0.0023
rs113382419	9	136463019	C	A	0.889	-0.041	0.003	3.00E-37	462,690	153.6	0.0018	0.0020
rs13296519	9	128471924	G	T	0.606	-0.014	0.002	8.10E-12	462,690	43.3	-0.0030	0.0020
rs1221148	9	122046875	C	G	0.587	0.013	0.002	7.30E-11	462,690	37.9	0.0005	0.0020
rs4543592	9	3014254	T	C	0.52	-0.012	0.002	4.50E-10	462,690	33.3	0.0006	0.0021
rs7039819	9	82430418	G	A	0.427	0.013	0.002	5.10E-10	462,690	38.3	0.0007	0.0024
rs1246265	9	86761745	T	C	0.305	-0.013	0.002	4.20E-09	462,690	33.2	0.0068	0.0029
rs12244388	10	104640052	G	A	0.661	-0.019	0.002	1.40E-19	462,690	74.9	0.0042	0.0025

rs3896224	10	106467853	A	G	0.585	0.014	0.002	1.10E-11	462,690	44.0	-0.0028	0.0022
rs11255908	10	8802912	T	G	0.743	-0.015	0.002	2.30E-10	462,690	39.8	-0.0021	0.0021
rs2675638	10	63576286	G	A	0.581	0.012	0.002	1.30E-09	462,690	32.4	-0.0067	0.0025
rs7077678	10	104438565	C	T	0.623	0.012	0.002	2.60E-09	462,690	31.3	-0.0037	0.0034
rs17553262	10	92912773	A	C	0.885	-0.018	0.003	5.30E-09	462,690	30.5	0.0049	0.0023
rs10823968	10	74738269	A	T	0.633	0.012	0.002	2.10E-08	462,690	31.0	0.0026	0.0036
rs9919670	11	112877304	G	A	0.612	-0.022	0.002	7.60E-27	462,690	106.4	-0.0004	0.0021
rs17309874	11	27667236	G	A	0.74	-0.016	0.002	9.70E-13	462,690	45.6	-0.0020	0.0058
rs4391802	11	28674592	A	G	0.707	0.015	0.002	1.40E-11	462,690	43.1	0.0046	0.0030
rs112282219	11	46632809	G	A	0.959	-0.033	0.005	3.80E-11	462,690	39.6	-0.0001	0.0056
rs75742406	11	17070365	G	A	0.739	0.014	0.002	1.30E-09	462,690	35.0	-0.0059	0.0022
rs34866095	11	16377356	A	G	0.686	-0.012	0.002	1.20E-08	462,690	28.7	-0.0008	0.0021
rs10879871	12	75380511	T	G	0.343	-0.014	0.002	5.00E-11	462,690	40.9	0.0030	0.0020
rs7297175	12	56473808	T	C	0.431	-0.012	0.002	6.60E-09	462,690	32.7	-0.0038	0.0020
rs12831617	12	84758368	C	T	0.764	-0.013	0.002	1.90E-08	462,690	28.2	0.0021	0.0021
rs74086911	12	50015942	G	A	0.925	0.021	0.004	2.10E-08	462,690	28.3	-0.0025	0.0023
rs7333559	13	100546450	G	A	0.212	0.015	0.002	3.20E-10	462,690	34.8	-0.0024	0.0021
rs6562474	13	67332812	C	G	0.651	0.012	0.002	1.00E-08	462,690	30.3	0.0094	0.0020
rs3742365	14	104198251	T	C	0.595	-0.016	0.002	2.50E-14	462,690	57.1	-0.0031	0.0020
rs7155595	14	77502546	A	C	0.674	-0.013	0.002	2.50E-09	462,690	34.4	0.0054	0.0027
rs860326	14	57342912	C	T	0.428	0.012	0.002	2.70E-09	462,690	32.6	-0.0023	0.0020
rs8042849	15	78817929	C	T	0.342	0.028	0.002	1.80E-39	462,690	163.3	0.0006	0.0021
rs35175834	15	47680815	G	A	0.788	-0.024	0.002	4.60E-22	462,690	89.1	0.0080	0.0025
rs8042134	15	97514404	T	G	0.541	-0.014	0.002	1.30E-12	462,690	45.0	-0.0017	0.0022
rs6598539	15	99204483	T	C	0.489	-0.012	0.002	4.50E-09	462,690	33.3	-0.0007	0.0032
rs28485305	15	74044197	C	T	0.631	0.012	0.002	2.60E-08	462,690	31.0	0.0180	0.0024
rs889398	16	69556715	C	T	0.588	0.013	0.002	6.30E-11	462,690	37.9	-0.0020	0.0027
rs369230	16	89645437	G	T	0.308	-0.013	0.002	1.80E-09	462,690	33.3	0.0008	0.0020
rs12708665	16	24728227	A	G	0.285	-0.013	0.002	3.50E-09	462,690	31.9	0.0012	0.0021
rs1050847	16	87443734	C	T	0.426	0.011	0.002	1.40E-08	462,690	27.4	-0.0003	0.0028
rs11861214	16	746611	G	T	0.784	0.014	0.002	2.00E-08	462,690	30.7	-0.0037	0.0024
rs60952428	16	75640521	T	C	0.909	0.019	0.003	3.00E-08	462,690	27.6	-0.0067	0.0038
rs57611503	16	31165795	G	A	0.485	0.011	0.002	4.00E-08	462,690	28.0	-0.0021	0.0021
rs8614	17	27588806	C	A	0.817	-0.017	0.003	1.80E-10	462,690	40.0	0.0010	0.0020
rs67596067	17	50333733	G	A	0.649	-0.013	0.002	1.20E-09	462,690	35.6	0.0011	0.0020
rs732083	17	37834367	G	A	0.333	0.012	0.002	1.50E-08	462,690	29.6	-0.0003	0.0022
rs9904288	17	47031973	T	C	0.708	0.012	0.002	3.10E-08	462,690	27.6	-0.0027	0.0023
rs71367545	18	77576337	G	A	0.791	-0.015	0.002	1.40E-09	462,690	34.4	0.0015	0.0051
rs62098013	18	50863861	G	A	0.64	-0.012	0.002	4.10E-09	462,690	30.7	-0.0020	0.0020

rs12967855	18	35138245	A	G	0.331	0.012	0.002	3.10E-08	462,690	29.5	-0.0015	0.0026
rs76608582	19	4474725	C	A	0.953	0.031	0.005	3.20E-10	462,690	39.8	0.0051	0.0020
rs35343344	19	18471610	C	A	0.733	0.013	0.002	8.80E-09	462,690	30.6	0.0010	0.0020
rs6011779	20	61984317	C	T	0.191	0.028	0.003	2.30E-27	462,690	112.1	-0.0060	0.0021
rs6119897	20	31145415	G	A	0.762	-0.018	0.002	3.60E-15	462,690	54.4	-0.0030	0.0020
rs4814873	20	19616429	C	T	0.767	0.014	0.002	2.90E-09	462,690	32.4	-0.0042	0.0026
rs12481282	20	44761377	G	C	0.722	-0.013	0.002	7.80E-09	462,690	31.4	-0.0075	0.0020
rs348809	20	59032097	A	G	0.348	-0.012	0.002	1.30E-08	462,690	30.2	0.0000	0.0021
rs2838834	21	46665208	C	T	0.699	-0.013	0.002	6.30E-10	462,690	32.9	0.0010	0.0021
rs147412694	21	40702786	G	A	0.85	-0.017	0.003	2.90E-09	462,690	34.1	0.0007	0.0021
rs202645	22	41798520	A	G	0.203	-0.015	0.002	3.90E-09	462,690	33.7	0.0007	0.0022
rs136233	22	31212410	A	G	0.809	-0.014	0.003	1.80E-08	462,690	28.0	0.0036	0.0020

SNP, single nucleotide polymorphism; Chr, chromosome; EA, effect allele; NEA, non-effect allele; EAF, effect allele frequency.

* N refers to the sample size of the initial GWAS from which the genetic variants were selected.

Table 3. Associations of single nucleotide polymorphisms for continuant sleep duration.

SNP	Chr	Position	EA	NEA	EAF	Beta	SE	*p*-value	N*	F-statistic	Outcome	
											Beta	SE
rs915416	1	34731984	C	G	0.61	0.0193	0.0025	9.90E-15	446,118	79.0	0.0001	0.0022
rs269054	1	57864304	T	A	0.56	-0.0136	0.0023	2.10E-09	446,118	41.0	0.0003	0.0020
rs61796569	1	66476437	C	T	0.20	-0.0154	0.0026	1.50E-09	446,118	33.5	-0.0003	0.0023
rs12567114	1	98527951	G	A	0.15	-0.0148	0.0025	4.30E-09	446,118	24.6	-0.0058	0.0023
rs62120041	2	9185564	T	C	0.48	0.0261	0.0046	9.60E-09	446,118	151.8	0.0062	0.0041
rs374153	2	40382712	C	T	0.16	0.0176	0.0031	9.10E-09	446,118	36.8	0.0006	0.0027
rs75539574	2	58871658	A	C	0.80	-0.0362	0.0041	6.90E-19	446,118	188.6	-0.0031	0.0037
rs72804080	2	59358659	A	G	0.95	-0.0178	0.0032	2.90E-08	446,118	13.5	-0.0068	0.0029
rs7556815	2	114085785	G	A	0.78	-0.0407	0.0027	1.30E-49	446,118	254.1	0.0008	0.0024
rs12611523	2	139195328	A	G	0.57	0.0126	0.0023	3.10E-08	446,118	34.9	0.0021	0.0020
rs4128364	2	147612734	T	C	0.84	-0.0146	0.0024	1.40E-09	446,118	26.0	-0.0002	0.0021
rs4538155	2	157040773	C	T	0.82	-0.0130	0.0024	3.60E-08	446,118	22.1	-0.0008	0.0021
rs11885663	2	166944004	C	T	0.92	-0.0162	0.0026	8.60E-10	446,118	17.8	0.0049	0.0023
rs10173260	2	210377845	T	C	0.29	-0.0128	0.0023	2.90E-08	446,118	30.3	-0.0108	0.0020
rs112230981	3	55879269	A	G	0.25	0.0315	0.0052	2.20E-09	446,118	165.4	0.0099	0.0047
rs17732997	3	70470834	C	G	0.46	0.0129	0.0023	1.20E-08	446,118	37.1	-0.0027	0.0020
rs7644809	3	107564459	T	C	0.28	0.0131	0.0023	1.60E-08	446,118	30.4	-0.0011	0.0020
rs13088093	3	135838598	T	G	0.74	-0.0163	0.0024	7.00E-12	446,118	45.7	0.0021	0.0021

rs2192528	4	18327896	A	G	0.52	0.0134	0.0023	2.70E-09	446,118	39.8	0.0000	0.0020
rs17427571	4	82254908	A	G	0.08	0.0138	0.0024	1.30E-08	446,118	12.7	0.0028	0.0022
rs35531607	4	92533225	T	C	0.34	-0.0128	0.0023	1.50E-08	446,118	32.8	0.0016	0.0020
rs13109404	4	102896591	T	G	0.93	0.0312	0.0044	1.40E-12	446,118	58.0	-0.0030	0.0040
rs365663	5	1428883	A	G	0.21	0.0146	0.0023	1.00E-10	446,118	31.3	-0.0050	0.0020
rs460692	5	3126584	C	T	0.58	0.0211	0.0033	3.60E-10	446,118	96.3	0.0019	0.0030
rs56372231	5	102321905	C	T	0.26	-0.0169	0.0024	2.20E-12	446,118	49.1	-0.0017	0.0021
rs11567976	5	137654218	C	T	0.31	-0.0128	0.0023	2.10E-08	446,118	31.5	0.0008	0.0020
rs151014368	5	176751059	G	A	0.57	-0.0161	0.0028	9.10E-09	446,118	56.7	0.0003	0.0025
rs34556183	6	28584775	A	G	0.87	0.0169	0.0025	2.30E-11	446,118	28.2	0.0160	0.0022
rs80193650	6	33464363	A	G	0.42	-0.0168	0.0031	4.10E-08	446,118	61.8	0.0026	0.0027
rs113113059	6	43160375	T	C	0.88	0.0161	0.0027	8.40E-09	446,118	24.6	0.0009	0.0024
rs9382445	6	54937974	T	C	0.77	0.0145	0.0023	4.80E-10	446,118	33.0	-0.0017	0.0021
rs2231265	6	89790201	A	G	0.38	-0.0150	0.0027	2.70E-08	446,118	47.2	-0.0034	0.0024
rs9345234	6	93162639	A	C	0.44	-0.0130	0.0023	1.80E-08	446,118	37.2	0.0023	0.0020
rs34731055	7	2106928	C	T	0.26	-0.0195	0.0029	3.70E-11	446,118	65.8	-0.0060	0.0026
rs2079070	7	114126432	C	G	0.75	0.0175	0.0026	7.50E-12	446,118	51.7	-0.0023	0.0023
rs7806045	7	132610266	T	C	0.48	0.0148	0.0026	1.40E-08	446,118	48.7	0.0056	0.0023
rs330088	8	9149746	T	C	0.77	-0.0145	0.0023	2.70E-10	446,118	32.8	-0.0004	0.0020
rs73219758	8	14279446	G	A	0.42	0.0164	0.0025	5.60E-11	446,118	58.6	-0.0003	0.0022
rs10973207	9	37100525	G	T	0.59	-0.0204	0.0031	6.00E-11	446,118	89.9	0.0065	0.0028
rs1776776	9	140497072	T	C	0.55	0.0200	0.0034	4.90E-09	446,118	88.1	NA	NA
rs12246842	10	21830580	A	G	0.71	0.0134	0.0023	3.90E-09	446,118	32.9	-0.0029	0.0020
rs10761674	10	64618340	C	T	0.72	0.0123	0.0023	4.20E-08	446,118	27.4	0.0049	0.0020
rs11190970	10	103128332	G	A	0.18	0.0154	0.0028	4.60E-08	446,118	31.3	0.0003	0.0025
rs7915425	10	125016501	T	C	0.47	0.0191	0.0030	2.00E-10	446,118	80.9	-0.0014	0.0027
rs1517572	11	28829882	A	C	0.55	-0.0146	0.0023	1.50E-10	446,118	47.4	0.0011	0.0020
rs4592416	11	43800474	A	G	0.16	-0.0147	0.0023	9.30E-11	446,118	25.6	-0.0013	0.0020
rs11602180	11	48162453	C	T	0.34	0.0182	0.0031	2.30E-09	446,118	66.5	0.0111	0.0027
rs174560	11	61581764	T	C	0.65	-0.0136	0.0024	2.80E-08	446,118	37.5	-0.0021	0.0022
rs12791153	11	80685181	A	T	0.46	-0.0235	0.0042	1.90E-08	446,118	123.1	0.0064	0.0038
rs1553132	11	88297740	A	G	0.14	-0.0145	0.0026	2.50E-08	446,118	22.3	-0.0030	0.0023
rs1939455	11	101520886	G	T	0.41	0.0204	0.0036	1.20E-08	446,118	90.3	0.0013	0.0031
rs7115226	11	113408518	C	A	0.64	-0.0266	0.0044	1.70E-09	446,118	144.3	-0.0012	0.0038
rs1263056	11	116576415	A	G	0.33	0.0128	0.0023	2.00E-08	446,118	32.5	-0.0052	0.0020
rs7951019	11	118358027	T	G	0.27	-0.0369	0.0065	1.20E-08	446,118	239.1	-0.0119	0.0059
rs1057703	11	122830251	T	G	0.14	-0.0194	0.0032	1.10E-09	446,118	41.2	0.0001	0.0028
rs34354917	12	38764559	C	A	0.93	0.0137	0.0025	3.90E-08	446,118	10.4	-0.0005	0.0022
rs4767550	12	117951150	A	G	0.07	-0.0143	0.0023	6.30E-10	446,118	12.4	0.0021	0.0021

rs6575005	14	26954078	T	C	0.15	0.0156	0.0026	4.40E-09	446,118	27.5	0.0019	0.0023
rs10483350	14	29816155	A	G	0.71	-0.0174	0.0029	1.50E-09	446,118	55.6	0.0018	0.0025
rs61985058	14	60233841	C	T	0.73	-0.0186	0.0032	1.30E-08	446,118	60.2	-0.0035	0.0029
rs55658675	14	65554638	C	T	0.09	0.0131	0.0024	2.00E-08	446,118	12.1	0.0198	0.0021
rs11621908	14	78495761	C	T	0.22	0.0241	0.0042	5.60E-09	446,118	88.7	-0.0077	0.0037
rs8038326	15	47989799	A	G	0.52	0.0159	0.0025	2.80E-10	446,118	56.4	-0.0087	0.0023
rs3095508	16	6550400	C	A	0.42	0.0154	0.0023	3.10E-11	446,118	51.3	0.0009	0.0020
rs11643715	16	23909538	C	G	0.75	-0.0139	0.0025	3.20E-08	446,118	31.9	-0.0049	0.0022
rs9940646	16	53800629	C	G	0.17	0.0169	0.0023	1.20E-13	446,118	36.9	0.0048	0.0020
rs8050478	16	56120461	G	A	0.03	0.0160	0.0023	1.70E-12	446,118	7.1	0.0008	0.0020
rs7503199	17	8134275	C	T	0.16	0.0147	0.0026	1.00E-08	446,118	26.4	0.0193	0.0023
rs205024	17	11227352	C	T	0.73	-0.0138	0.0023	3.90E-09	446,118	33.9	0.0023	0.0021
rs1991556	17	44083402	G	A	0.29	0.0166	0.0027	1.00E-09	446,118	50.4	-0.0034	0.0024
rs9903973	17	50571227	C	T	0.58	0.0128	0.0023	2.60E-08	446,118	35.5	-0.0008	0.0020
rs12607679	18	53059748	T	C	0.62	0.0201	0.0026	8.30E-15	446,118	85.0	-0.0029	0.0023
rs10421649	19	9942262	T	A	0.47	-0.0133	0.0023	6.90E-09	446,118	39.3	0.0024	0.0020
rs2072727	20	43538733	T	C	0.58	0.0132	0.0023	7.90E-09	446,118	38.2	-0.0026	0.0020

SNP, single nucleotide polymorphism; Chr, chromosome; EA, effect allele; NEA, non-effect allele; EAF, effect allele frequency.

* N refers to the sample size of the initial GWAS from which the genetic variants were selected.

Table 4. Associations of single nucleotide polymorphisms for short sleep duration.

SNP	Chr	Position	EA	NEA	EAF	Beta	SE	*p*-value	N*	F-statistic	Outcome	
											Beta	SE
rs7524118	1	34736052	C	T	0.71	0.03	0.0065	4.90E-08	411,934	215.4	-0.0002	0.0022
rs2820313	1	201870221	G	A	0.34	0.031	0.006	2.30E-09	411,934	117.0	-0.0045	0.0021
rs2186122	1	66470206	T	A	0.56	0.024	0.006	4.80E-09	411,934	177.7	0.0009	0.0020
rs12567114	1	98527951	G	A	0.72	0.036	0.0064	4.10E-09	411,934	152.7	-0.0058	0.0023
rs1380703	2	57941287	G	A	0.07	0.035	0.0059	1.60E-11	411,934	65.7	-0.0007	0.0021
rs2863957	2	114089551	C	A	0.78	0.054	0.0073	2.60E-18	411,934	412.7	0.0008	0.0024
rs75539574	2	58871658	A	C	0.91	0.045	0.0108	8.40E-11	411,934	136.7	-0.0031	0.0037
rs2014830	3	50172397	C	T	0.7	0.03	0.0065	2.70E-08	411,934	155.8	-0.0084	0.0022
rs13107325	4	103188709	T	C	0.07	0.075	0.011	2.50E-13	411,934	301.9	-0.0001	0.0039
rs17005118	4	82288564	A	G	0.26	0.03	0.0065	2.50E-09	411,934	142.7	-0.0030	0.0023
rs12518468	5	7249696	C	T	0.66	0.031	0.0065	8.50E-09	411,934	177.7	0.0003	0.0021
rs3776864	5	102327868	A	C	0.67	0.031	0.0065	1.70E-08	411,934	175.1	-0.0021	0.0021
rs4585442	5	135508381	G	A	0.31	0.031	0.006	8.10E-10	411,934	169.4	0.0020	0.0022
rs12661667	6	41792545	T	C	0.26	0.028	0.0065	2.80E-08	411,934	124.3	0.0022	0.0023
rs9321171	6	129848635	C	T	0.54	0.031	0.006	4.20E-08	411,934	196.8	-0.0033	0.0020
rs9367621	6	55040290	T	A	0.43	0.024	0.0055	1.60E-08	411,934	116.3	0.0001	0.0020
rs11763750	7	2080114	G	A	0.81	0.035	0.0079	5.10E-09	411,934	155.4	-0.0063	0.0026
rs1229762	7	114218582	T	C	0.66	0.037	0.0064	1.00E-12	411,934	253.3	0.0014	0.0021
rs60882754	8	52886619	A	T	0.94	0.055	0.0122	1.80E-08	411,934	140.6	-0.0055	0.0041
rs1607227	11	28808617	G	T	0.7	0.031	0.0065	1.50E-09	411,934	166.3	-0.0012	0.0022
rs7939345	11	47980568	T	G	0.21	0.035	0.0074	4.00E-08	411,934	167.5	-0.0105	0.0025
rs17388803	15	48027204	C	A	0.11	0.053	0.0098	6.50E-10	411,934	226.7	0.0045	0.0033
rs59779556	16	56227965	T	G	0.55	0.025	0.006	2.00E-08	411,934	127.5	-0.0011	0.0020
rs205024	17	11227352	C	T	0.62	0.031	0.0065	2.70E-08	411,934	186.6	0.0023	0.0021
rs12963463	18	53099093	C	T	0.26	0.029	0.0065	1.90E-11	411,934	133.4	0.0009	0.0022
rs5757675	22	39838892	G	T	0.26	0.034	0.0069	2.70E-09	411,934	183.3	-0.0017	0.0023

SNP, single nucleotide polymorphism; Chr, chromosome; EA, effect allele; NEA, non-effect allele; EAF, effect allele frequency.

* N refers to the sample size of the initial GWAS from which the genetic variants were selected.

Table 5. Associations of single nucleotide polymorphisms for long sleep duration.

SNP	Chr	Position	EA	NEA	EAF	Beta	SE	*p*-value	N*	F-statistic	Outcome	
											Beta	SE
rs7534398	1	7767464	A	T	0.20	0.0469	0.0118	2.10E-08	339,926	240.5	0.0065	0.0025
rs6737318	2	114083120	G	A	0.22	0.0760	0.0110	3.40E-13	339,926	679.9	-0.0009	0.0024
rs10899257	11	76415209	A	G	0.14	0.0677	0.0130	4.60E-08	339,926	385.1	0.0021	0.0028
rs3751046	11	122828342	G	A	0.15	0.0695	0.0135	2.00E-08	339,926	413.4	-0.0003	0.0028
rs75458655	11	118115331	T	C	0.02	0.1848	0.0292	5.40E-12	339,926	522.0	-0.0117	0.0066
rs17817288	16	53807764	A	G	0.52	0.0392	0.0094	8.90E-09	339,926	261.3	0.0039	0.0020
rs17688916	17	43778680	T	A	0.80	0.0714	0.0125	1.10E-11	339,926	563.0	-0.0020	0.0026

SNP, single nucleotide polymorphism; Chr, chromosome; EA, effect allele; NEA, non-effect allele; EAF, effect allele frequency.

* N refers to the sample size of the initial GWAS from which the genetic variants were selected.

Table 6. Associations of single nucleotide polymorphisms for insomnia.

SNP	Chr	Position	EA	NEA	EAF	Beta	SE	P-value	N*	F-statistic	Outcome	
											Beta	SE
rs699844	1	74878253	A	G	0.92	0.0602	0.0110	4.11E-08	1,331,010	710	-0.0028	0.0036
rs1620977	1	72729142	A	G	0.27	0.0516	0.0070	2.27E-14	1,331,010	1398	0.0016	0.0023
rs10800992	1	190900576	T	C	0.44	0.0421	0.0060	3.84E-12	1,331,010	1164	-0.0034	0.0020
rs12030482	1	96961268	A	T	0.22	0.0411	0.0070	8.16E-09	1,331,010	772	-0.0019	0.0024
rs5877	1	173878862	T	C	0.67	0.0363	0.0060	1.23E-08	1,331,010	776	0.0006	0.0021
rs11588755	1	57819204	A	G	0.52	-0.0346	0.0060	5.14E-09	1,331,010	796	-0.0008	0.0020
rs11119409	1	210293333	T	C	0.59	-0.0346	0.0060	1.19E-08	1,331,010	771	0.0009	0.0020
rs6702604	1	107190062	A	G	0.58	-0.0367	0.0060	1.30E-09	1,331,010	874	0.0022	0.0020
rs623025	1	201765094	T	C	0.26	-0.0377	0.0070	3.16E-08	1,331,010	728	0.0010	0.0023
rs1937447	1	66358242	C	G	0.76	-0.0387	0.0070	2.08E-08	1,331,010	728	0.0007	0.0024
rs2089358	1	37194103	T	C	0.7	-0.0408	0.0070	2.75E-10	1,331,010	931	-0.0018	0.0022
rs1289939	1	117944435	T	C	0.23	-0.0408	0.0070	6.00E-09	1,331,010	785	0.0042	0.0024
rs11803128	1	190060095	A	G	0.65	-0.0408	0.0060	6.85E-11	1,331,010	1009	0.0019	0.0021
rs113851554	2	66750564	T	G	0.05	0.2062	0.0140	1.56E-51	1,331,010	5398	-0.0072	0.0045
rs62158170	2	114082175	A	G	0.79	0.0658	0.0070	1.20E-19	1,331,010	1915	0.0016	0.0024
rs75452188	2	67134426	A	G	0.88	0.0516	0.0090	1.58E-08	1,331,010	749	-0.0063	0.0031
rs116466468	2	159137557	T	C	0.76	0.044	0.0070	2.11E-10	1,331,010	941	0.0025	0.0024
rs55772859	2	208042581	A	C	0.31	0.0421	0.0060	4.82E-11	1,331,010	1010	0.0005	0.0022
rs56097173	2	44262449	T	C	0.68	0.0402	0.0060	2.69E-10	1,331,010	937	-0.0020	0.0021
rs12991815	2	68071990	C	G	0.42	0.0402	0.0060	3.02E-11	1,331,010	1049	0.0011	0.0020

rs1861412	2	58893065	A	G	0.43	0.0383	0.0060	1.67E-10	1,331,010	958	-0.0074	0.0020
rs11679943	2	77724624	A	G	0.35	0.0373	0.0060	3.16E-09	1,331,010	843	0.0000	0.0021
rs6756610	2	147480394	C	G	0.63	0.0373	0.0060	1.14E-09	1,331,010	864	0.0000	0.0021
rs62213452	2	210380152	T	G	0.28	0.0373	0.0070	2.39E-08	1,331,010	747	-0.0150	0.0023
rs1530938	2	236900633	A	G	0.44	0.0363	0.0060	8.82E-10	1,331,010	865	-0.0015	0.0020
rs34967082	2	215382654	A	G	0.41	0.0354	0.0060	4.34E-09	1,331,010	807	-0.0011	0.0020
rs72820274	2	104412924	A	G	0.42	0.0344	0.0060	1.28E-08	1,331,010	768	0.0023	0.0021
rs10928256	2	146458738	T	C	0.42	0.0344	0.0060	1.61E-08	1,331,010	768	-0.0008	0.0020
rs823247	2	2850540	T	C	0.48	-0.0367	0.0060	5.25E-10	1,331,010	896	-0.0013	0.0020
rs1519102	2	66677816	C	G	0.69	-0.0367	0.0060	1.90E-08	1,331,010	767	-0.0027	0.0022
rs7599697	2	239231477	T	C	0.36	-0.0367	0.0060	5.00E-09	1,331,010	827	0.0014	0.0021
rs7571486	2	176473295	A	G	0.25	-0.0387	0.0070	1.40E-08	1,331,010	748	0.0001	0.0023
rs6545798	2	60521311	A	T	0.41	-0.0408	0.0060	1.19E-11	1,331,010	1073	0.0020	0.0021
rs4664299	2	160570033	T	C	0.23	-0.0408	0.0070	4.95E-09	1,331,010	785	0.0007	0.0023
rs6734957	2	42813247	T	G	0.24	-0.0419	0.0070	1.82E-09	1,331,010	853	-0.0050	0.0024
rs13010288	2	51824512	T	G	0.13	-0.0598	0.0090	9.26E-12	1,331,010	1078	0.0103	0.0030
rs62264767	3	117642005	A	C	0.85	0.0649	0.0080	1.63E-14	1,331,010	1431	-0.0008	0.0028
rs10865954	3	49211989	T	C	0.33	0.0421	0.0060	1.92E-11	1,331,010	1044	0.0000	0.0021
rs17025198	3	88001713	A	G	0.2	0.0411	0.0070	2.19E-08	1,331,010	720	-0.0030	0.0025
rs6808140	3	10581380	T	C	0.51	0.0392	0.0060	5.35E-11	1,331,010	1023	0.0011	0.0020
rs35110063	3	43066558	A	G	0.43	0.0392	0.0060	8.82E-11	1,331,010	1003	-0.0005	0.0020
rs7625896	3	44062561	A	G	0.65	0.0363	0.0060	5.28E-09	1,331,010	798	-0.0035	0.0021
rs2216427	3	180785697	C	G	0.65	0.0354	0.0060	1.60E-08	1,331,010	759	-0.0005	0.0021
rs4260410	3	178469932	T	C	0.33	0.0344	0.0060	4.87E-08	1,331,010	697	-0.0068	0.0021
rs1567084	3	71435955	A	G	0.5	0.0334	0.0060	2.14E-08	1,331,010	743	-0.0020	0.0020
rs1580173	3	107955515	A	G	0.56	0.0334	0.0060	2.28E-08	1,331,010	732	-0.0002	0.0020
rs4858708	3	25154112	A	T	0.53	-0.0336	0.0060	1.23E-08	1,331,010	749	0.0015	0.0020
rs2364921	3	158522463	T	C	0.47	-0.0336	0.0060	2.13E-08	1,331,010	749	0.0005	0.0020
rs7615602	3	18718055	C	G	0.27	-0.0398	0.0070	2.59E-09	1,331,010	832	0.0073	0.0022
rs3774751	3	50209053	T	G	0.46	-0.0408	0.0060	7.32E-12	1,331,010	1102	0.0121	0.0020
rs694786	3	173112907	T	C	0.46	-0.044	0.0060	1.97E-13	1,331,010	1281	0.0023	0.0020
rs492858	3	155432229	T	C	0.08	-0.0661	0.0110	3.46E-09	1,331,010	857	-0.0001	0.0037
rs17005118	4	82288564	A	G	0.26	0.0421	0.0070	6.13E-10	1,331,010	908	-0.0030	0.0023
rs11722569	4	112822731	T	C	0.66	0.0344	0.0060	2.91E-08	1,331,010	707	-0.0031	0.0021
rs13138995	4	148987430	A	G	0.39	0.0344	0.0060	1.97E-08	1,331,010	750	-0.0021	0.0021
rs62301574	4	22050165	C	G	0.8	-0.0419	0.0070	1.37E-08	1,331,010	748	0.0002	0.0025
rs16990210	4	34720226	T	C	0.85	-0.046	0.0080	1.97E-08	1,331,010	719	-0.0030	0.0028
rs72657797	4	90820809	T	C	0.18	-0.0555	0.0080	1.52E-12	1,331,010	1211	0.0015	0.0027
rs4699157	4	106055212	T	C	0.96	-0.0812	0.0150	3.98E-08	1,331,010	674	-0.0002	0.0050

rs13135092	4	103198082	A	G	0.92	-0.0888	0.0110	2.53E-16	1,331,010	1547	0.0011	0.0037
rs55972276	5	135653737	A	C	0.14	0.0733	0.0090	4.19E-17	1,331,010	1724	-0.0007	0.0029
rs16903122	5	87693561	T	C	0.25	0.0554	0.0070	9.04E-16	1,331,010	1534	0.0021	0.0023
rs17223714	5	50492629	A	G	0.79	0.0459	0.0070	2.44E-10	1,331,010	931	0.0021	0.0025
rs35539975	5	91607148	A	G	0.78	0.0421	0.0070	4.49E-09	1,331,010	810	-0.0006	0.0024
rs12187443	5	102660400	T	C	0.67	0.0402	0.0060	1.64E-10	1,331,010	952	-0.0016	0.0021
rs6888135	5	141254063	A	C	0.5	0.0383	0.0060	1.21E-10	1,331,010	977	0.0033	0.0020
rs6601080	5	179511043	A	G	0.68	0.0354	0.0060	2.21E-08	1,331,010	726	0.0014	0.0021
rs12520974	5	61514611	T	C	0.48	-0.0356	0.0060	1.69E-09	1,331,010	843	-0.0001	0.0020
rs701394	5	80296487	A	G	0.64	-0.0356	0.0060	6.83E-09	1,331,010	778	0.0006	0.0021
rs17367725	5	107112116	T	C	0.35	-0.0356	0.0060	9.29E-09	1,331,010	768	0.0005	0.0021
rs4502882	5	153093998	T	C	0.66	-0.0387	0.0060	7.96E-10	1,331,010	895	0.0002	0.0021
rs17083297	5	92995477	A	C	0.18	-0.044	0.0080	1.60E-08	1,331,010	761	0.0003	0.0026
rs152555	5	106849674	A	G	0.85	-0.0523	0.0080	4.83E-10	1,331,010	929	-0.0038	0.0029
rs2431108	5	103947968	T	C	0.67	-0.0534	0.0060	7.83E-17	1,331,010	1680	-0.0020	0.0021
rs8180457	5	107209814	T	C	0.16	-0.0555	0.0080	1.12E-11	1,331,010	1103	0.0069	0.0028
rs62383308	5	165460085	A	G	0.08	-0.0598	0.0110	3.98E-08	1,331,010	701	-0.0023	0.0037
rs11756035	6	18843810	C	G	0.13	0.0507	0.0090	1.29E-08	1,331,010	774	0.0004	0.0030
rs62429521	6	140324582	A	C	0.15	0.0507	0.0080	1.78E-09	1,331,010	873	-0.0030	0.0028
rs9373590	6	101212001	A	T	0.51	0.0402	0.0060	2.18E-11	1,331,010	1076	-0.0019	0.0020
rs1147852	6	147980909	A	G	0.31	0.0392	0.0060	9.94E-10	1,331,010	876	0.0007	0.0022
rs10947987	6	41754370	T	C	0.44	-0.0325	0.0060	4.08E-08	1,331,010	693	0.0019	0.0020
rs728017	6	124292594	A	G	0.39	-0.0346	0.0060	9.51E-09	1,331,010	759	0.0019	0.0021
rs2388840	6	99598756	A	G	0.58	-0.0367	0.0060	1.37E-09	1,331,010	874	-0.0024	0.0020
rs10944696	6	94498850	A	G	0.3	-0.0377	0.0070	7.99E-09	1,331,010	795	0.0033	0.0022
rs6457796	6	34828553	T	C	0.73	-0.0387	0.0070	1.12E-08	1,331,010	786	0.0060	0.0023
rs314281	6	105400605	T	C	0.45	-0.0429	0.0060	6.03E-13	1,331,010	1214	-0.0023	0.0020
rs3131638	6	31475127	A	G	0.23	-0.044	0.0070	7.88E-10	1,331,010	913	0.0093	0.0024
rs10947690	6	37631768	A	G	0.74	-0.0471	0.0070	4.04E-12	1,331,010	1137	0.0031	0.0023
rs9394502	6	38452503	T	C	0.33	-0.0545	0.0060	7.76E-18	1,331,010	1751	0.0011	0.0021
rs4709655	6	163280204	T	C	0.12	-0.0545	0.0090	3.09E-09	1,331,010	835	0.0023	0.0031
rs10947428	6	33647058	T	C	0.79	-0.0683	0.0070	9.06E-21	1,331,010	2063	0.0052	0.0024
rs138678612	6	30932223	A	G	0.98	-0.1165	0.0200	1.41E-08	1,331,010	709	-0.0131	0.0066
rs6465151	7	88310899	T	C	0.11	0.0564	0.0090	1.90E-09	1,331,010	830	0.0023	0.0031
rs670501	7	108625185	T	C	0.21	0.0526	0.0070	7.40E-13	1,331,010	1223	-0.0043	0.0024
rs12666306	7	115082406	A	G	0.5	0.0421	0.0060	2.24E-12	1,331,010	1181	-0.0040	0.0020
rs2598293	7	133989882	T	C	0.48	0.0354	0.0060	2.48E-09	1,331,010	833	-0.0008	0.0020
rs6978112	7	1966841	T	C	0.41	0.0344	0.0060	2.11E-08	1,331,010	762	-0.0010	0.0020
rs2030672	7	21687925	C	G	0.56	0.0344	0.0060	1.10E-08	1,331,010	777	-0.0001	0.0020

rs190073	7	10985188	A	G	0.41	-0.0336	0.0060	2.86E-08	1,331,010	727	0.0014	0.0020
rs1731951	7	137075847	A	T	0.44	-0.0346	0.0060	1.36E-08	1,331,010	786	0.0047	0.0020
rs521484	7	49894349	A	G	0.77	-0.0398	0.0070	1.53E-08	1,331,010	747	0.0064	0.0024
rs75932578	7	106844694	T	C	0.22	-0.0398	0.0070	4.15E-08	1,331,010	724	0.0001	0.0025
rs6967168	7	132672192	T	G	0.75	-0.044	0.0070	1.39E-10	1,331,010	967	0.0048	0.0023
rs8180817	7	114047542	C	G	0.43	-0.0492	0.0060	1.83E-16	1,331,010	1581	-0.0009	0.0020
rs73671843	7	3520024	A	G	0.13	-0.0555	0.0090	5.49E-10	1,331,010	928	0.0024	0.0030
rs17520265	7	119674508	A	G	0.03	-0.091	0.0160	2.87E-08	1,331,010	642	0.0140	0.0055
rs28611339	8	10170037	T	G	0.13	0.0583	0.0090	8.46E-11	1,331,010	1024	0.0038	0.0030
rs2737240	8	116657235	A	G	0.71	0.0363	0.0070	3.37E-08	1,331,010	723	-0.0009	0.0022
rs871994	8	35190619	A	C	0.44	0.0354	0.0060	5.50E-09	1,331,010	822	0.0001	0.0020
rs874168	8	30849450	T	C	0.53	0.0344	0.0060	7.95E-09	1,331,010	785	-0.0028	0.0020
rs4588900	8	73890425	A	G	0.52	0.0334	0.0060	1.57E-08	1,331,010	742	-0.0234	0.0020
rs28552587	8	103356226	A	G	0.56	0.0334	0.0060	3.30E-08	1,331,010	732	-0.0032	0.0020
rs10955647	8	114154187	T	G	0.53	0.0334	0.0060	1.84E-08	1,331,010	740	-0.0006	0.0020
rs671985	8	60914783	A	G	0.45	-0.0377	0.0060	2.79E-10	1,331,010	937	0.0014	0.0020
rs17643634	8	91650818	T	C	0.17	-0.0598	0.0080	1.34E-13	1,331,010	1345	0.0028	0.0027
rs77641763	9	140265782	T	C	0.12	0.0714	0.0090	6.53E-15	1,331,010	1435	NA	NA
rs118166957	9	8858043	T	C	0.16	0.0677	0.0080	1.95E-16	1,331,010	1642	-0.0008	0.0027
rs2792990	9	125621610	C	G	0.86	0.0545	0.0080	1.15E-10	1,331,010	953	-0.0012	0.0028
rs1927902	9	120518991	T	C	0.25	0.0526	0.0070	1.15E-14	1,331,010	1382	-0.0005	0.0023
rs7040224	9	134886837	A	G	0.32	0.0373	0.0060	4.24E-09	1,331,010	806	0.0030	0.0021
rs72773790	9	139109080	T	C	0.67	0.0373	0.0060	3.71E-09	1,331,010	819	-0.0020	0.0022
rs10756571	9	14534505	T	C	0.69	0.0363	0.0060	1.80E-08	1,331,010	751	-0.0039	0.0022
rs6597649	9	133786652	T	C	0.4	0.0334	0.0060	3.05E-08	1,331,010	713	0.0015	0.0020
rs10758593	9	4292083	A	G	0.4	-0.0356	0.0060	4.90E-09	1,331,010	810	0.0032	0.0020
rs4090240	9	77118987	T	C	0.28	-0.0387	0.0070	8.46E-09	1,331,010	804	0.0005	0.0023
rs7044885	9	81739348	C	G	0.44	-0.0408	0.0060	5.67E-12	1,331,010	1093	-0.0007	0.0020
rs10761240	9	96361922	A	G	0.4	-0.0429	0.0060	2.12E-12	1,331,010	1177	-0.0079	0.0021
rs10825503	10	57177470	T	G	0.49	0.0334	0.0060	1.43E-08	1,331,010	743	0.0005	0.0020
rs7475916	10	77771194	C	G	0.35	-0.0367	0.0060	6.70E-09	1,331,010	816	0.0012	0.0021
rs11001276	10	76825638	A	T	0.74	-0.0377	0.0070	2.52E-08	1,331,010	728	-0.0022	0.0023
rs12251016	10	21821918	A	T	0.66	-0.0387	0.0060	3.89E-10	1,331,010	895	-0.0051	0.0021
rs224029	10	64519299	T	C	0.4	-0.0387	0.0060	2.51E-10	1,331,010	958	0.0056	0.0020
rs1064939	11	118396331	A	T	0.98	0.1302	0.0200	2.16E-10	1,331,010	885	-0.0185	0.0069
rs72899452	11	45415577	T	C	0.06	0.0742	0.0120	1.00E-09	1,331,010	827	-0.0060	0.0040
rs56133505	11	72348039	A	G	0.54	0.0411	0.0060	5.59E-12	1,331,010	1118	0.0005	0.0020
rs4592425	11	62697813	T	G	0.7	0.0402	0.0060	4.31E-10	1,331,010	904	0.0064	0.0022
rs2221119	11	88598444	C	G	0.44	0.0363	0.0060	2.00E-09	1,331,010	865	-0.0014	0.0020

rs10898940	11	73455292	A	C	0.52	0.0344	0.0060	8.09E-09	1,331,010	787	-0.0033	0.0020
rs667730	11	83277325	T	C	0.58	0.0334	0.0060	2.26E-08	1,331,010	724	-0.0027	0.0020
rs647905	11	121534938	T	C	0.54	0.0334	0.0060	2.87E-08	1,331,010	738	-0.0016	0.0020
rs214934	11	17193475	A	T	0.31	-0.0377	0.0060	3.16E-09	1,331,010	810	0.0027	0.0022
rs6589988	11	99126016	A	G	0.68	-0.0377	0.0060	4.70E-09	1,331,010	824	0.0018	0.0021
rs566673	11	66401373	T	G	0.54	-0.0387	0.0060	1.18E-10	1,331,010	991	0.0049	0.0020
rs12790660	11	57667222	T	C	0.68	-0.0398	0.0060	4.49E-10	1,331,010	918	0.0046	0.0022
rs524859	11	66041079	A	G	0.36	-0.044	0.0060	1.48E-12	1,331,010	1188	0.0013	0.0021
rs11605348	11	47606483	A	G	0.35	-0.045	0.0060	7.01E-13	1,331,010	1227	0.0028	0.0021
rs2286729	12	6873818	A	G	0.09	0.0695	0.0110	5.37E-11	1,331,010	1054	-0.0105	0.0036
rs12310246	12	84700945	A	G	0.25	0.045	0.0070	4.74E-11	1,331,010	1012	-0.0068	0.0023
rs6606731	12	109982578	A	T	0.19	0.0431	0.0080	1.51E-08	1,331,010	761	-0.0051	0.0025
rs324017	12	57487814	A	C	0.29	0.0392	0.0070	1.61E-09	1,331,010	843	0.0016	0.0022
rs1167132	12	43484487	T	C	0.39	0.0354	0.0060	8.73E-09	1,331,010	794	0.0032	0.0020
rs4767645	12	118385788	T	G	0.46	-0.0367	0.0060	6.47E-10	1,331,010	891	-0.0023	0.0020
rs61921611	12	66367726	T	C	0.69	-0.044	0.0060	7.84E-12	1,331,010	1103	0.0041	0.0022
rs28582096	12	123856998	A	G	0.21	-0.0545	0.0070	1.74E-13	1,331,010	1313	-0.0184	0.0025
rs7992992	13	54721699	A	G	0.13	0.0507	0.0090	1.15E-08	1,331,010	774	-0.0028	0.0030
rs11149313	13	85294881	A	G	0.73	0.0402	0.0070	2.38E-09	1,331,010	848	-0.0007	0.0022
rs6562066	13	60532796	T	C	0.37	0.0392	0.0060	1.38E-10	1,331,010	954	-0.0019	0.0021
rs9540729	13	66947124	A	T	0.48	0.0363	0.0060	1.40E-09	1,331,010	876	-0.0003	0.0020
rs9563886	13	61720066	T	C	0.61	-0.0336	0.0060	3.08E-08	1,331,010	715	-0.0009	0.0020
rs1536053	13	111982291	T	C	0.32	-0.0377	0.0060	6.04E-09	1,331,010	824	0.0001	0.0022
rs2389631	13	96932868	A	C	0.67	-0.0398	0.0060	2.03E-10	1,331,010	933	0.0001	0.0021
rs1031654	13	54382035	A	C	0.8	-0.0513	0.0070	3.88E-12	1,331,010	1122	0.0047	0.0025
rs9527083	13	53991125	A	G	0.67	-0.0758	0.0060	1.61E-32	1,331,010	3390	0.0023	0.0021
rs4981170	14	33412996	A	G	0.19	-0.0545	0.0080	7.33E-13	1,331,010	1218	-0.0034	0.0025
rs7168238	15	66709386	C	G	0.07	0.0639	0.0110	1.80E-08	1,331,010	708	-0.0023	0.0038
rs715338	15	57215867	A	G	0.58	0.0411	0.0060	7.85E-12	1,331,010	1096	0.0013	0.0020
rs1038093	15	74012409	T	C	0.63	0.0392	0.0060	2.47E-10	1,331,010	954	0.0007	0.0021
rs176644	15	89913632	T	G	0.4	0.0354	0.0060	9.49E-09	1,331,010	801	-0.0015	0.0020
rs7402939	15	99183876	T	C	0.38	-0.0356	0.0060	5.19E-09	1,331,010	795	0.0008	0.0021
rs12917449	15	74331659	A	C	0.81	-0.0419	0.0080	2.97E-08	1,331,010	720	0.0143	0.0026
rs12912299	15	38897857	T	C	0.49	-0.0429	0.0060	4.42E-13	1,331,010	1225	0.0044	0.0020
rs4702	15	91426560	A	G	0.56	-0.0481	0.0060	6.78E-16	1,331,010	1519	0.0072	0.0020
rs1015438	16	51177517	A	G	0.19	0.0583	0.0080	2.51E-14	1,331,010	1394	-0.0026	0.0026
rs35322724	16	77137324	A	C	0.58	0.0488	0.0060	3.75E-16	1,331,010	1546	-0.0012	0.0020
rs9931543	16	56128782	T	C	0.74	0.0478	0.0070	1.11E-12	1,331,010	1171	0.0022	0.0023
rs3902952	16	61647589	T	C	0.19	0.0478	0.0080	2.55E-10	1,331,010	937	0.0016	0.0026

rs830716	16	12323509	C	G	0.71	0.045	0.0070	8.68E-12	1,331,010	1111	0.0018	0.0022
rs34214423	16	52303107	A	C	0.81	0.045	0.0080	3.18E-09	1,331,010	830	-0.0002	0.0026
rs12924275	16	9191790	T	C	0.27	0.0383	0.0070	1.93E-08	1,331,010	770	0.0062	0.0023
rs4788203	16	29978827	A	G	0.43	-0.0346	0.0060	6.32E-09	1,331,010	782	0.0008	0.0020
rs3184470	16	715164	A	G	0.35	-0.0377	0.0060	9.73E-10	1,331,010	861	-0.0004	0.0021
rs4238755	16	52746089	A	C	0.26	-0.0429	0.0070	2.30E-10	1,331,010	943	-0.0033	0.0023
rs66674044	16	19904344	A	T	0.86	-0.0598	0.0090	2.18E-12	1,331,010	1147	-0.0012	0.0029
rs11650304	17	46035001	C	G	0.93	0.0667	0.0120	1.23E-08	1,331,010	771	-0.0010	0.0040
rs34490907	17	26933741	C	G	0.89	0.0535	0.0090	1.76E-08	1,331,010	746	-0.0040	0.0032
rs62068188	17	2400876	T	C	0.83	0.0488	0.0080	1.18E-09	1,331,010	895	-0.0002	0.0027
rs4643373	17	47123423	T	C	0.7	0.0411	0.0070	1.58E-10	1,331,010	945	0.0008	0.0022
rs8076183	17	61024696	T	C	0.45	-0.0377	0.0060	2.75E-10	1,331,010	937	-0.0044	0.0020
rs9889282	17	50259142	A	C	0.61	-0.0419	0.0060	4.70E-12	1,331,010	1113	-0.0003	0.0021
rs7214267	17	43157709	A	G	0.58	-0.044	0.0060	5.09E-13	1,331,010	1257	-0.0004	0.0020
rs12605642	18	31313965	T	G	0.49	0.0354	0.0060	2.13E-09	1,331,010	834	0.0012	0.0020
rs9964420	18	56824041	A	C	0.3	0.0354	0.0070	4.54E-08	1,331,010	701	-0.0017	0.0022
rs12454003	18	26315799	C	G	0.48	-0.0346	0.0060	4.94E-09	1,331,010	796	-0.0005	0.0020
rs10502966	18	50748499	A	G	0.58	-0.0387	0.0060	8.54E-11	1,331,010	972	0.0003	0.0021
rs60565673	18	52906830	T	G	0.62	-0.0429	0.0060	1.59E-12	1,331,010	1155	-0.0031	0.0021
rs908668	19	56134038	T	C	0.21	0.0497	0.0070	1.41E-11	1,331,010	1092	0.0008	0.0025
rs429358	19	45411941	T	C	0.85	0.0459	0.0080	2.13E-08	1,331,010	715	-0.0173	0.0028
rs6510033	19	30710785	A	G	0.73	-0.0367	0.0070	4.66E-08	1,331,010	707	-0.0008	0.0022
rs12983032	19	5073447	A	G	0.34	-0.0429	0.0060	1.07E-11	1,331,010	1100	0.0040	0.0021
rs742760	20	50985290	A	T	0.82	0.0431	0.0080	2.48E-08	1,331,010	730	-0.0006	0.0026
rs2867690	20	41972028	T	C	0.18	0.0421	0.0080	3.70E-08	1,331,010	697	-0.0027	0.0026
rs6019663	20	47774512	T	C	0.29	0.0402	0.0070	6.47E-10	1,331,010	886	-0.0031	0.0022
rs910187	20	45841052	A	G	0.37	-0.0346	0.0060	1.63E-08	1,331,010	743	-0.0045	0.0021
rs76145129	20	62670427	T	G	0.12	-0.0502	0.0090	2.73E-08	1,331,010	709	-0.0118	0.0031
rs6119267	20	31163914	C	G	0.69	-0.0598	0.0060	2.32E-20	1,331,010	2039	0.0058	0.0022
rs2838787	21	46539725	A	G	0.39	-0.0356	0.0060	7.65E-09	1,331,010	803	0.0035	0.0021
rs11090039	22	41496800	A	G	0.29	0.0392	0.0070	1.82E-09	1,331,010	843	-0.0079	0.0022

SNP, single nucleotide polymorphism; Chr, chromosome; EA, effect allele; NEA, non-effect allele; EAF, effect allele frequency.

* N refers to the sample size of the initial GWAS from which the genetic variants were selected.

Table 7. Associations of single nucleotide polymorphisms for moderate-to-vigorous physical activity.

SNP	Chr	Position	EA	NEA	EAF	Beta	SE	*p*-value	N*	F-statistic	Outcome	
											Beta	SE
rs2035562	3	85,056,521	A	G	0.33	-0.0140	0.0020	3.90E-09	377,234	32.7	-0.0016	0.0020
rs2854277	6	32,628,084	C	T	0.92	0.0320	0.0050	2.60E-10	377,234	56.9	0.0414	0.0050
rs3094622	6	30,327,952	A	G	0.86	0.0200	0.0030	1.40E-09	377,234	36.3	0.0253	0.0030
rs149943	6	28,002,388	G	A	0.85	0.0190	0.0030	2.20E-09	377,234	34.7	0.0216	0.0030
rs7804463	7	133,447,651	T	C	0.53	0.0150	0.0020	1.20E-11	377,234	42.3	-0.0015	0.0020
rs7791992	7	50,237,784	C	A	0.41	-0.0140	0.0020	5.70E-10	377,234	35.8	0.0038	0.0020
rs1043595	7	128,410,012	G	A	0.72	0.0140	0.0020	4.30E-09	377,234	29.8	-0.0016	0.0020
rs2988004	9	37,044,388	T	G	0.56	-0.0130	0.0020	4.10E-09	377,234	31.4	-0.0065	0.0020
rs429358	19	45,411,941	T	C	0.85	-0.0220	0.0030	6.10E-13	377,234	46.6	-0.0173	0.0030

SNP, single nucleotide polymorphism; Chr, chromosome; EA, effect allele; NEA, non-effect allele; EAF, effect allele frequency.

[*] N refers to the sample size of the initial GWAS from which the genetic variants were selected.

Table 8. Associations of single nucleotide polymorphisms for vigorous physical activity.

SNP	Chr	Position	EA	NEA	EAF	Beta	SE	*p*-value	N*	F-statistic	Outcome	
											Beta	SE
rs1248860	3	85,015,779	G	A	0.48	-0.0408	0.0055	1.10E-13	261,055	217.1	-0.0018	0.0020
rs2764261	6	108,927,842	A	G	0.37	0.0392	0.0058	2.00E-11	261,055	187.1	0.0015	0.0021
rs328902	7	35,020,843	C	T	0.69	-0.0408	0.0066	5.50E-10	261,055	186.0	0.0026	0.0022
rs13243553	7	133,506,955	G	A	0.61	0.0392	0.006	9.00E-11	261,055	191.0	-0.0017	0.0021
rs3781411	10	126,715,436	C	T	0.88	0.0583	0.0093	3.00E-10	261,055	187.5	-0.0025	0.0030

SNP, single nucleotide polymorphism; Chr, chromosome; EA, effect allele; NEA, non-effect allele; EAF, effect allele frequency.

* N refers to the sample size of the initial GWAS from which the genetic variants were selected.

Table 9. Associations of single nucleotide polymorphisms for strenuous sports or other exercises.

SNP	Chr	Position	EA	NEA	EAF	Beta	SE	*p*-value	N*	F-statistic	Outcome	
											Beta	SE
rs62253088	3	85,400,801	T	C	0.33	0.0488	0.0059	1.00E-19	350,492	369.5	0.0037	0.0021
rs159544	5	60,489,247	A	G	0.61	-0.0305	0.0050	1.30E-09	350,492	155.2	-0.0030	0.0020
rs111901094	5	19,513,570	G	T	0.82	0.0392	0.0066	3.00E-09	350,492	159.1	-0.0013	0.0026
rs10946808	6	26233387	A	G	0.73	-0.0305	0.0050	9.90E-10	350,492	128.6	0.0010	0.0023
rs75930676	14	71,826,547	T	C	0.95	-0.0726	0.0121	2.00E-09	350,492	175.6	0.0064	0.0047
rs166840	17	19,799,698	G	A	0.59	0.0296	0.0045	3.10E-11	350,492	148.6	0.0005	0.0021

SNP, single nucleotide polymorphism; Chr, chromosome; EA, effect allele; NEA, non-effect allele; EAF, effect allele frequency.

* N refers to the sample size of the initial GWAS from which the genetic variants were selected.

Table 10. Post-hoc power calculations for our main IVW analyses on lifestyle factors and telomere length.

Lifestyle factors	Sample size*	R^2 of exposure by genetic variants†	Causal effect (OR according to IVW)	Significance level	Power‡
Lifetime smoking	472,174	0.0110	0.882	0.01	100%
Insomnia	472,174	0.0260	0.972	0.01	100%
Sleep duration	472,174	0.0069	1.059	0.01	100%
MVPA	472,174	0.0009	1.680	0.01	100%

* Sample size according to the outcome GWAS on telomere length.

†Variance explained of exposure by genetic variants as reported in GWASs on the different lifestyle factors.

‡ Calculated using an online power calculation tool (https://sb452.shinyapps.io/power/).

MVPA: Moderate-to-vigorous physical activity; OR: True odds ratio of the outcome variable per standard deviation of the exposure variable.

Table 11. Estimates for the association between lifestyle factors and telomere length.

**Outcome**	**Method**	**OR**	**95% CI**	***p*-value**
Lifetime smoking	IVW (multiplicative random effects)	0.882	(0.847, 0.918)	9.22E-10
	Weighted median	0.882	(0.846, 0.920)	5.87E-09
	MR Egger	0.839	(0.715, 0.985)	0.034
Insomnia	IVW (multiplicative random effects)	0.972	(0.959, 0.985)	1.76E-05
	Weighted median	0.980	(0.968, 0.991)	5.34E-04
	MR Egger	0.984	(0.934, 1.038)	0.563
Sleep duration	IVW (multiplicative random effects)	1.059	(0.984, 1.140)	0.123
	Weighted median	0.990	(0.938, 1.044)	0.704
	MR Egger	0.987	(0.749, 1.301)	0.928
MVPA	IVW (multiplicative random effects)	1.680	(1.115, 2.531)	0.013
	Weighted median	1.080	(0.867, 1.345)	0.493
	MR Egger	11.934	(4.167, 34.180)	0.002

MVPA, Moderate-to-vigorous physical activity; OR, Odds ratio; CI, Confidence interval.

Table 12. Heterogeneity and MR-Egger test for Horizontal pleiotropy

**Exposure**	**Outcomes**	**Heterogeneity**			
		**Q**	**O_df**	***p*-value**	**I² (%)**
Lifetime smoking	Telomere length	290.7	124	2.02E-15	57.3%
Insomnia		675.3	204	8.17E-52	69.8%
Sleep duration		372.3	71	2.08E-42	80.9%
MVPA		49.2	7	2.09E-08	85.8%
		**MR-Egger test for directional pleiotropy**			
		**Egger_intercept**	**Standard error**	***p*-value**	
Lifetime smoking	Telomere length	0.001	0.001	0.532	
Insomnia		-0.001	0.001	0.626	
Sleep duration		0.001	0.002	0.605	
MVPA		-0.033	0.009	0.007	

MVPA, Moderate-to-vigorous physical activity.

Table 13. Associations between genetic liability to lifestyle factors and telomere length following exclusion of outlier SNPs identified by MR-PRESSO.

Outcome	Exposures	SNP	IVW<br>OR (95% CI)	*p*-value	MR PRESSO<br>OR (95%CI)	*p*-value
Telomere length	Lifetime smoking	126	0.882 (0.847, 0.918)	9.22E-10	0.882 (0.846, 0.919)[1]	1.91E-09
	Insomnia	207	0.972 (0.959, 0.985)	1.76E-05	0.974 (0.965, 0.983)[2]	2.31E-08
	Sleep duration	74	1.059 (0.984, 1.140)	0.123	1.061 (0.982, 1.147)[3]	0.134
	MVPA	9	1.680 (1.115, 2.531)	0.013	2.482 (1.171, 5.263)[4]	0.018

MVPA, Moderate-to-vigorous physical activity; SNP, single nucleotide polymorphism; IVW, the inverse-variance weighted method; OR, odds ratio; CI, confidence interval; MR-PRESSO, MR-Pleiotropy Residual Sum and Outlier.

[1] MR-PRESSO IV outliers detected: rs7039819, rs35343344, rs1050847.

[2] MR-PRESSO IV outliers detected: rs10761240, rs12917449, rs28582096, rs3774751, rs429358, rs4588900, rs4592425, rs62213452, rs76145129.

[3] MR-PRESSO IV outliers detected: rs915416, rs1776776, rs1553132, rs10483350, rs8050478.

[4] MR-PRESSO IV outliers detected: rs429358, rs7804463, rs7791992, rs149943, rs2988004, rs1043595.

The code used in the current study

```
rm(list=ls())
library(TwoSampleMR)
ao <- available_outcomes()

exposure_dat = clump_data(exposure_dat,
                       clump_kb = 10000,
                       clump_r2 = 0.01,
                       clump_p1 = 1,
                       clump_p2 = 1,
                       pop = "EUR")

exposure_dat <-read_exposure_data(
  filename = 'Sleep/Insomnia-207.csv',
  sep = ',',
  snp_col = 'SNP',
  beta_col = 'beta',
  se_col = 'se',
  effect_allele_col = 'effect_allele',
  phenotype_col = 'Phenotype',
  units_col = 'units',
  other_allele_col = 'other_allele',
  eaf_col = 'eaf',
  samplesize_col = 'samplesize',
  ncase_col = 'ncase',
  ncontrol_col = 'ncontrol',
  gene_col = 'gene',
  pval_col = 'pval'
)
exposure_dat$exposure="insomnia"

exposure_dat <-read_exposure_data(
  filename = 'Physical activity/MVPA-9.csv',
  sep = ',',
  snp_col = 'SNP',
  beta_col = 'beta',
  se_col = 'se',
  effect_allele_col = 'effect_allele',
  phenotype_col = 'Phenotype',
  units_col = 'units',
  other_allele_col = 'other_allele',
  eaf_col = 'eaf',
```

```
  samplesize_col = 'samplesize',
  ncase_col = 'ncase',
  ncontrol_col = 'ncontrol',
  gene_col = 'gene',
  pval_col = 'pval'
)
exposure_dat$exposure="Physical activity"

exposure_dat <-read_exposure_data(
  filename = 'Sleep/Sleep duration-74.csv',
  sep = ',',
  snp_col = 'SNP',
  beta_col = 'beta',
  se_col = 'se',
  effect_allele_col = 'effect_allele',
  phenotype_col = 'Phenotype',
  units_col = 'units',
  other_allele_col = 'other_allele',
  eaf_col = 'eaf',
  samplesize_col = 'samplesize',
  ncase_col = 'ncase',
  ncontrol_col = 'ncontrol',
  gene_col = 'gene',
  pval_col = 'pval'
)
exposure_dat$exposure="Sleep duration"

exposure_dat <-read_exposure_data(
  filename = 'Sleep/Long sleep duration-7.csv',
  sep = ',',
  snp_col = 'SNP',
  beta_col = 'beta',
  se_col = 'se',
  effect_allele_col = 'effect_allele',
  phenotype_col = 'Phenotype',
  units_col = 'units',
  other_allele_col = 'other_allele',
  eaf_col = 'eaf',
  samplesize_col = 'samplesize',
  ncase_col = 'ncase',
  ncontrol_col = 'ncontrol',
  gene_col = 'gene',
  pval_col = 'pval'
```

```
)
exposure_dat$exposure="Long sleep duration"

exposure_dat <-read_exposure_data(
  filename = 'Sleep/Short sleep duration-26.csv',
  sep = ',',
  snp_col = 'SNP',
  beta_col = 'beta',
  se_col = 'se',
  effect_allele_col = 'effect_allele',
  phenotype_col = 'Phenotype',
  units_col = 'units',
  other_allele_col = 'other_allele',
  eaf_col = 'eaf',
  samplesize_col = 'samplesize',
  ncase_col = 'ncase',
  ncontrol_col = 'ncontrol',
  gene_col = 'gene',
  pval_col = 'pval'
)
exposure_dat$exposure="Short sleep duration"

exposure_dat <-read_exposure_data(
  filename = 'Risk factor/Life smoking-126.csv',
  sep = ',',
  snp_col = 'SNP',
  beta_col = 'beta',
  se_col = 'se',
  effect_allele_col = 'effect_allele',
  phenotype_col = 'Phenotype',
  units_col = 'units',
  other_allele_col = 'other_allele',
  eaf_col = 'eaf',
  samplesize_col = 'samplesize',
  ncase_col = 'ncase',
  ncontrol_col = 'ncontrol',
  gene_col = 'gene',
  pval_col = 'pval'
)
exposure_dat$exposure="Lifetime smoking index"

exposure_dat <-read_exposure_data(
  filename = 'Physical activity/VPA-5.csv',
```

```
  sep = ',',
  snp_col = 'SNP',
  beta_col = 'beta',
  se_col = 'se',
  effect_allele_col = 'effect_allele',
  phenotype_col = 'Phenotype',
  units_col = 'units',
  other_allele_col = 'other_allele',
  eaf_col = 'eaf',
  samplesize_col = 'samplesize',
  ncase_col = 'ncase',
  ncontrol_col = 'ncontrol',
  gene_col = 'gene',
  pval_col = 'pval'
)
exposure_dat$exposure="Vigorous physical activity"

exposure_dat <-read_exposure_data(
  filename = 'Physical activity/SSOE-6.csv',
  sep = ',',
  snp_col = 'SNP',
  beta_col = 'beta',
  se_col = 'se',
  effect_allele_col = 'effect_allele',
  phenotype_col = 'Phenotype',
  units_col = 'units',
  other_allele_col = 'other_allele',
  eaf_col = 'eaf',
  samplesize_col = 'samplesize',
  ncase_col = 'ncase',
  ncontrol_col = 'ncontrol',
  gene_col = 'gene',
  pval_col = 'pval'
)
exposure_dat$exposure="strenuous sports or other exercises"


Telomere <- read_outcome_data(
  snps = exposure_dat$SNP,
  filename = "E:/MR Dataset/Telomere length/Telomere_length.txt",
  "\t",
  snp_col = "variant_id",
  beta_col = "beta",
```

```
  se_col = "standard_error",
  effect_allele_col = "effec_allele",
  other_allele_col = "other_allele",
  pval_col = "p_value")
Telomere$outcome = "Telomere length"


dat <- harmonise_data(exposure_dat,Telomere,action = 1)

mr_results <- mr(dat,method_list=c("mr_ivw_mre",
                                 "mr_weighted_median",
                                 "mr_egger_regression"
                                 ))

OR_mr_results=generate_odds_ratios(mr_results)

write.table(OR_mr_results, file = "results/dat.txt", sep = "\t", row.names = F, quote = F)


het=mr_heterogeneity(dat)
ple=mr_pleiotropy_test(dat)
write.table(het, file = "results/het.txt", sep = "\t",row.names = F, quote = F)
write.table(ple, file = "results/ple.txt", sep = "\t",row.names = F, quote = F)


library(MRPRESSO)
mr_presso(BetaOutcome = "beta.outcome", BetaExposure = "beta.exposure",
          SdOutcome = "se.outcome", SdExposure = "se.exposure",
          OUTLIERtest = TRUE, DISTORTIONtest = TRUE, data = dat,
          NbDistribution = 1000,  SignifThreshold = 0.05)


#----------------MVMR-------------
library(MendelianRandomization)
rm(list=ls())

exposure_dat <-read_exposure_data(
  filename = 'Sleep/Insomnia-207.csv',
  sep = ',',
  snp_col = 'SNP',
  beta_col = 'beta',
  se_col = 'se',
  effect_allele_col = 'effect_allele',
```

```
  phenotype_col = 'Phenotype',
  units_col = 'units',
  other_allele_col = 'other_allele',
  eaf_col = 'eaf',
  samplesize_col = 'samplesize',
  ncase_col = 'ncase',
  ncontrol_col = 'ncontrol',
  gene_col = 'gene',
  pval_col = 'pval'
)
exposure_dat$exposure="insomnia"

exposure_dat <-read_exposure_data(
  filename = 'Risk factor/Life smoking-126.csv',
  sep = ',',
  snp_col = 'SNP',
  beta_col = 'beta',
  se_col = 'se',
  effect_allele_col = 'effect_allele',
  phenotype_col = 'Phenotype',
  units_col = 'units',
  other_allele_col = 'other_allele',
  eaf_col = 'eaf',
  samplesize_col = 'samplesize',
  ncase_col = 'ncase',
  ncontrol_col = 'ncontrol',
  gene_col = 'gene',
  pval_col = 'pval'
)
exposure_dat$exposure="Lifetime smoking index"

BMI <- extract_outcome_data(
  snps = exposure_dat$SNP,
  outcomes = 'ieu-a-2')
BMI$outcome = "Body mass index"

Alcohol <- extract_outcome_data(
  snps = exposure_dat$SNP,
  outcomes = 'ieu-b-73')
Alcohol$outcome = "Alcohol"

AUD <- extract_outcome_data(
  snps = exposure_dat$SNP,
```

```
  outcomes = 'finn-b-AUD_SWEDISH')
AUD$outcome = "Alcohol disorder"

T2D <- extract_outcome_data(
  snps = exposure_dat$SNP,
  outcomes = 'ieu-a-23')
T2D$outcome = "Type 2 diabetes"

Lifetime_smoking <- read_outcome_data(
  snps = Telomere$SNP,
  filename = "E:/MR Dataset/Lifetime smoking index/lifetime smoking index.txt",
  "\t",
  snp_col = "SNP",
  beta_col = "BETA",
  se_col = "SE",
  effect_allele_col = "EFFECT_ALLELE",
  other_allele_col = "OTHER_ALLELE",
  pval_col = "P")

insomnia <- read_outcome_data(
  snps = exposure_dat$SNP,
  filename = "E:/MR Dataset/Insomnia/Insomnia_Jansenetal-248.txt",
  "\t",
  snp_col = "SNP",
  beta_col = "OR",
  se_col = "SE",
  effect_allele_col = "A1",
  other_allele_col = "A2",
  pval_col = "P",
  chr_col = "CHR",
  pos_col = "BP",
  eaf_col = "MAF"
)

Telomere <- read_outcome_data(
  snps = Lifetime_smoking$SNP,
  filename = "E:/MR Dataset/Telomere length/Telomere_length.txt",
  "\t",
  snp_col = "variant_id",
  beta_col = "beta",
  se_col = "standard_error",
  effect_allele_col = "effec_allele",
  other_allele_col = "other_allele",
```

```
  pval_col = "p_value")
Telomere$outcome = "Telomere length"


dat_exp <- harmonise_data(exposure_dat,Lifetime_smoking, action = 1)

dat_out <-harmonise_data(exposure_dat,Telomere, action = 1)

{GY <- dat_out$beta.outcome
  GY_SE <- dat_out$se.outcome
  GX_adj <- dat_exp$beta.outcome
  Gx_SE_adj <- dat_exp$se.outcome
  GX_exp <- dat_out$beta.exposure
  Gx_SE_exp <- dat_out$se.exposure
}

MRInputObject <- mr_mvinput(bx = cbind(GX_exp,GX_adj),
                            bxse = cbind(Gx_SE_exp,Gx_SE_adj),
                            by = GY,
                            byse = GY_SE)

tem= mr_mvivw(MRInputObject, model = "random",
              correl = FALSE,distribution = "normal", alpha = 0.05)

tem
```
